# Angiogenin and Osteoprotegerin are type II muscle specific myokines protecting pancreatic beta-cells against proinflammatory cytokines

**DOI:** 10.1038/s41598-018-28117-2

**Published:** 2018-07-03

**Authors:** Sabine Rutti, Rodolphe Dusaulcy, Jakob S. Hansen, Cédric Howald, Emmanouil T. Dermitzakis, Bente K. Pedersen, Michel Pinget, Peter Plomgaard, Karim Bouzakri

**Affiliations:** 1 UMR DIATHEC, EA 7294, Centre Européen d’Etude du Diabète, Université de Strasbourg, Fédération de Médecine Translationnelle de Strasbourg (FMTS), Bld René Leriche, 67200 Strasbourg, France; 2 Molecular Diabetes Laboratory, Division of Endocrinology-Diabetes-Hypertension and Nutrition, University Hospital/University of Geneva Medical School, 1211 Geneva, Switzerland; 3 Department of Clinical Biochemistry, Rigshospitalet, Copenhagen, Denmark; 4 Centre of Physical Activity Research, Rigshospitalet, Copenhagen, Denmark; 5 Department of Genetic Medicine and Development, University of Geneva Medical School, 1211 Geneva, Switzerland; 6 Institute for Genetics and Genomics in Geneva (iGE3), University of Geneva, 1211 Geneva, Switzerland; 7 Swiss Institute of Bioinformatics, Geneva, Switzerland

## Abstract

Tissue cross-talk is emerging as a determinant way to coordinate the different organs implicated in glucose homeostasis. Among the inter-organ communication factors, muscle-secreted myokines can modulate the function and survival of pancreatic beta-cells. Using primary human myotubes from soleus, vastus lateralis and triceps brachii muscles, we report here that the impact of myokines on beta-cells depends on fiber types and their metabolic status. We show that Type I and type II primary myotubes present specific mRNA and myokine signatures as well as a different sensitivity to TNF-alpha induced insulin resistance. Finally, we show that angiogenin and osteoprotegerin are triceps specific myokines with beta-cell protective actions against proinflammatory cytokines. These results suggest that type I and type II muscles could impact insulin secretion and beta-cell mass differentially in type 2 diabetes through specific myokines secretion.

## Introduction

Type 2 diabetes has a complex pathophysiology implicating several organs such as the adipose tissue, the liver, muscles and the pancreas. Nevertheless, pancreatic beta-cells and the skeletal muscle can be considered as a central hinge of this disease.

The muscle-pancreas axis was described for the first time by our group in 2011^1^. In this study, we established a model in which primary beta-cells were treated with conditioned medium prepared from human primary myotubes obtained from vastus lateralis biopsies. We reported that human skeletal muscle cells produce and release myokines depending on their state of insulin sensitivity, with bimodal action depending on insulin resistance of the skeletal muscle cells used to condition culture medium^1,2^. Nevertheless, although all skeletal muscles share the same contractile function, they cannot be considered a homogenous organ from a metabolic point of view. The human body contains about 600 skeletal muscles, which can be classified in three main groups. Type I muscles (e.g. soleus) are mainly composed of type I fibers that are characterized by a slow ATP consumption rate and an oxidative metabolism able to generate enough ATP to cover energy needs during a long exercise^3^. Type II muscles (e.g. triceps brachii) are mainly composed of type II fibers and are highly fatigable. Type II fibers have a rate limiting step of glycolytic metabolism and therefore cannot generate enough ATP to cover the high ATP consuming rate of myosin heavy chain II during exercise of long duration^3^. The last group is composed of muscles containing an approximately equivalent amount of type I and type II fibers (e.g. vastus lateralis)^4^.

In the present work, we have established human *in vitro* models of skeletal muscle cells isolated from type I and type II muscles and study their sensitivity to TNF-alpha induced insulin resistance. We have then investigated how the muscle type influences the profile of myokines secretion and their impact on beta-cells in order to identify new myokines implicated in fiber type specific muscle pancreas crosstalk. We show here for the first time, that skeletal muscle cells from biopsies with different fiber type composition present a unique gene expression and myokine signature. Moreover, the effect of human skeletal muscle cells on pancreatic beta-cells is fiber type specific, with both positive and negative effects depending on the level of insulin sensitivity. Finally we show that angiogenin (ANG) and osteoprotegerin (OPG) are triceps specific myokines that reduce apoptosis of beta-cells. These 2 myokines also prevent the apoptosis induced either by pro-inflammatory cytokines (cytomix: TNF-alpha, INFgamma and IL-1beta) or the negative effect of insulin resistant conditioned medium from soleus skeletal muscle cells (TNF-S-CM). Morevover, OPG counteracts both the cytomix and TNF-S-CM negative effects on primary pancreatic beta-cells proliferation and insulin secretion.

## Results

### RNA sequencing (RNA-seq) approach reveals a unique signature in cells isolated from soleus and triceps biopsies

**Figure 1:**
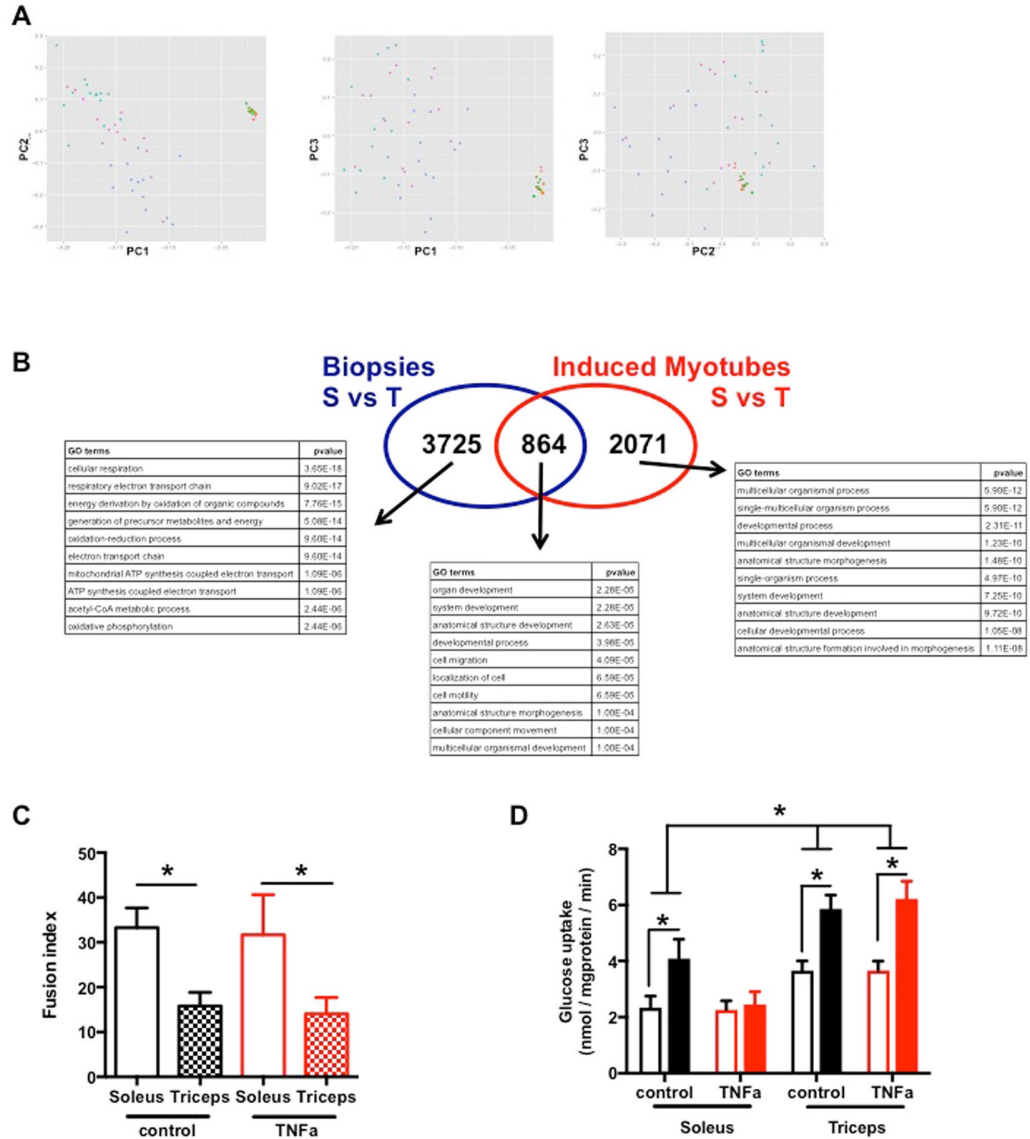
Soleus and triceps have different gene expression profiles and phenotypes. (**A**) Principal component analysis of the RPKM values of all genes with a RPKM >= 1 in at least 90% of the samples. Only biopsies were kept. Principal component 2 (PC2 axis) separates the soleus (HM-S) form the triceps (HM-T) whereas the vastus (HM-V) is spread all over PC2. (**B**) The Venn diagram shows the number of differentially expressed genes between the soleus (S) and the triceps (T) in both biopsies and differentiated myotubes. The 10 first most significant GO terms are displayed and sorted by their adjusted p-values (the full list of GO terms and KEGG pathways can be found in the Supplementary Table 1). (**C**) Fusion index of SKMC determined as the number of nucleus in the fiber. SKMC were cultured for 24 h in the presence or not of TNF-alpha (20 ng/ml); n = 6. (**D**) Glucose uptake was measured on SKMC under basal conditions (open box) and after insulin stimulation (100 nM) (closed box). Cells were pretreated for 24 h with TNF-alpha (20 ng/ml); n = 6. *p < 0.05 for indicated comparisons as tested by ANOVA followed by Bonferroni post hoc test.

In order to characterize the transcriptomes of biopsies and primary differentiated myotubes from soleus, triceps and vastus muscle, we established gene expression profiles using RNA-seq. The correlation of the overall gene expression within biopsies or myotubes is very high (spearman rho ~0.9) whereas it drops when comparing the biopsies with the myotubes (spearman rho ~0.5) (Supplementary Fig. 1). A principal component analysis (PCA) on RPKM values segregates well the biopsies from the differentiated myotubes (Fig. 1A, PC1). The soleus and the triceps biopsies form two distinct clusters while the vastus is more spread. This probably reflects the heterogeneous structure of this muscle type composed of both type I and II fibers (Supplementary Fig. 2, PC1 and PC2). The separation between the soleus and the triceps in induced myotubes is less evident probably because of the incomplete differentiation of the cultured cells (Supplementary Fig. 3). The comparison between induced myotubes MC-S and MC-T shows 2935 differentially expressed genes that hit gene ontology terms and KEGG pathways such as extracellular region, developmental process, focal adhesion and cytokine-cytokine receptor. However, 864 genes are differentially expressed both in the biopsies and in the myotubes (Fig. 1B). The gene ontology analysis on these genes highlights pathways such as organ development and developmental process that could reflect the common differentiation pathways occurring in biopsies and induced myotubes when stem cells are differentiating into soleus or triceps. Our results show that myotubes isolated from soleus and triceps present a different genes signature.

### MC-S and MC-T exhibit different phenotypes and respond differently to TNF-alpha

We first evaluated the myogenic capacity of each muscle cells preparation to differentiate into multinuclear myotubes. MC-T and MC-S were stained for myosin heavy chain and DAPI and fusion index was determined as the number of nucleus in the fiber. MC-S showed a higher rate of fusion compared to MC-T both in control conditions and in the presence of TNF-alpha (Fig. 1C). To monitor the capacity of TNF-alpha to induce insulin resistance (IR) in each muscle cells population, we measured glucose uptake under control conditions and after 24 h TNF-alpha treatment (Fig. 1D). In the absence of TNF-alpha, basal and insulin induced glucose uptake in MC-T is increased compared to MC-S. As previously observed in cells isolated from vastus lateralis biopsies^5^, TNF-alpha induces insulin resistance in MC-S. Interestingly, TNF-alpha failed to induce insulin resistance in MC-T.

### MC-S and MC-T have different myokine profiles under control conditions and after TNF-alpha treatment

**Figure 2:**
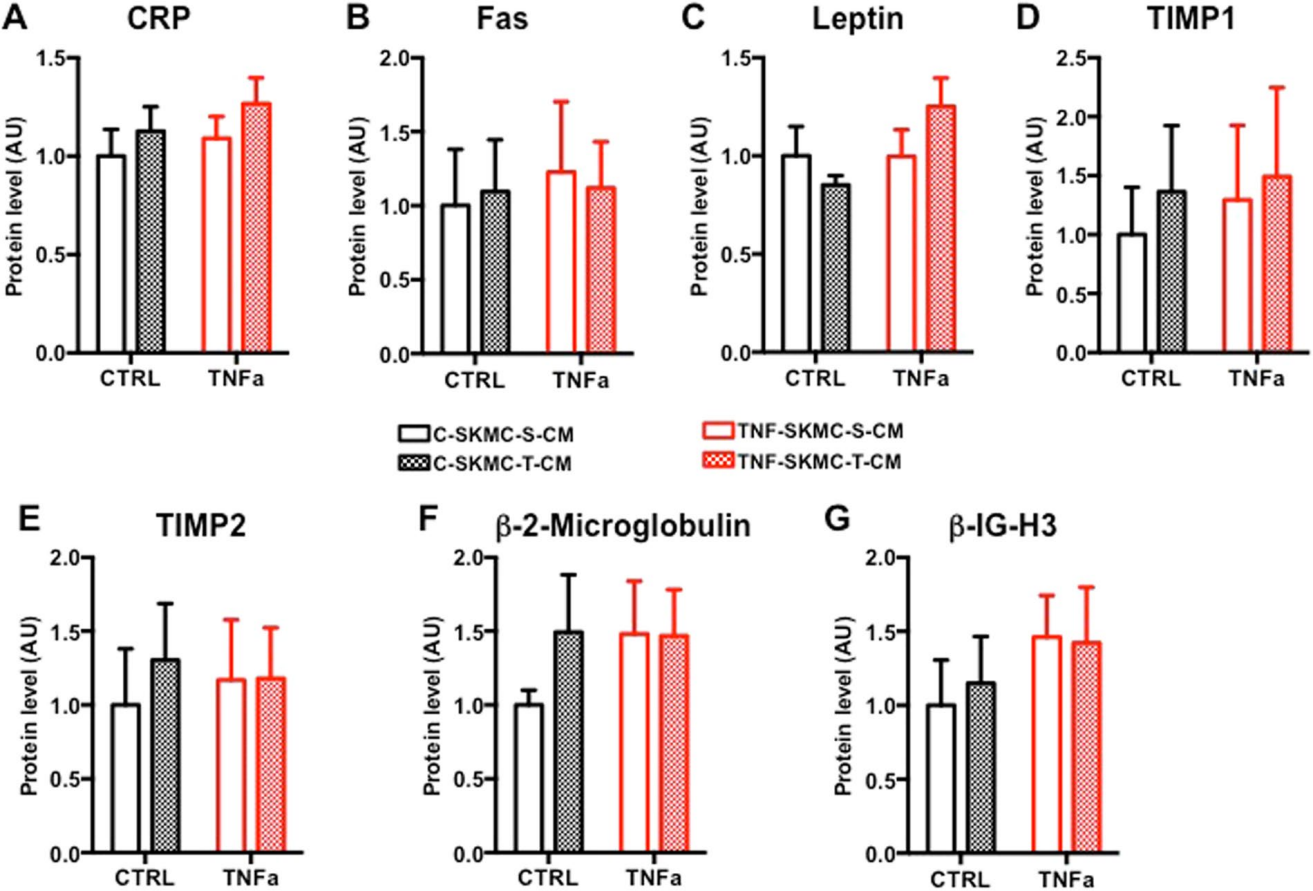
SKMC-S and SKMC-T have different myokine profiles under control conditions and after TNF-alpha treatment. Cytokines secreted in the conditioned media of SKMC-S (open bars) and SKMC-T (closed bars) cells in control condition or in the presence of TNF-alpha in the medium for 24 h, n = 4. C-reactive protein (CRP; (**A**)); Fas ligand (Fas; (**B**)), Leptin (**C**), Tissue Inhibitor of Metalloproteinases 1 (TIMP1;(**D**)), Tissue Inhibitor of Metalloproteinases 2 (TIMP2; (**E**)), beta-2-microglobulin (**F**), Transforming growth factor beta-induced (b-IG-H3; (**G**)).

**Figure 3:**
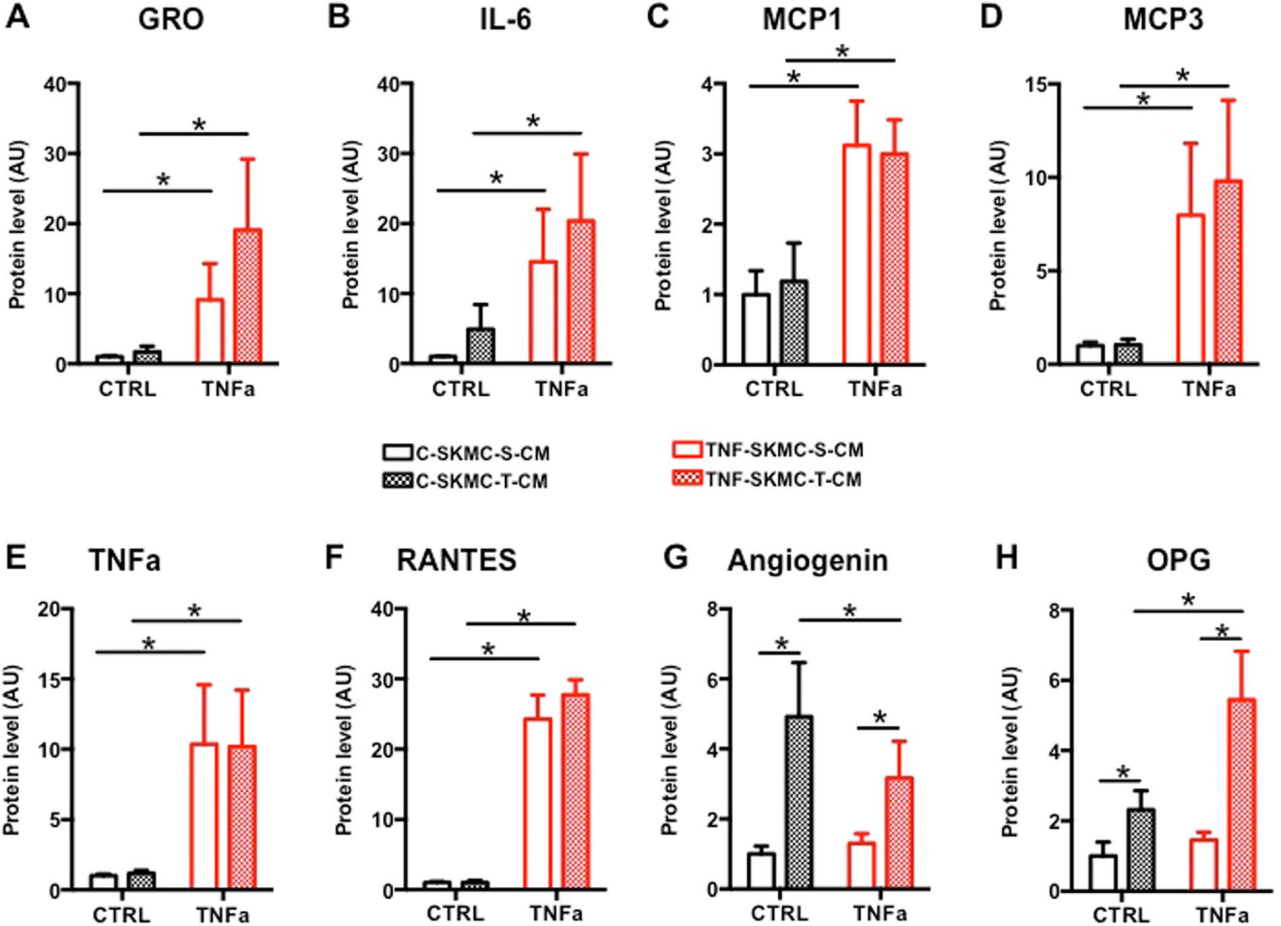
SKMC-S and SKMC-T have different myokine profiles under control conditions and after TNF-alpha treatment. Cytokines secreted in the conditioned media of SKMC-S (open bars) and SKMC-T (closed bars) cells in control condition or in the presence of TNF-alpha in the medium for 24 h, n = 4. GRO1 Oncogene (GRO; (**A**)); Interleukin-6 (IL-6; (**B**)), Monocyte Chemotactic Protein 1 (MCP1; (**C**)), Monocyte Chemotactic Protein 3 (MCP3; (**D**)), Regulated on Activation, Normal T Cell Expressed and Secreted (RANTES; (**E**)), Tumor necrosis factor alpha (TNFa; (**F**)), Angiogenin (**G**), osteoprotegerin (OPG; (**H**)). *p < 0.05 for the indicated comparisons as tested by ANOVA followed by Bonferroni post hoc test.

In order to analyze the secretome profile of each muscle cells population, we performed cytokine assays. This method allowed us to detect the presence of 119 different cytokines (Supplementary Table 2) secreted in the conditioned medium (CM) of MC-S and MC-T cells during control condition and in the presence of TNF-alpha in the medium for 24 h (Figs 2 and 3). Throughout this manuscript, CM from TNF-alpha treated muscle cells will be noted as TNF-S/T-CM and control CM will be noted as C-S/T-CM. TNF-alpha detection can be considered as a positive control since it was exogenously added to the medium during the treatment. Leptin, Fas, TIMP1 and TIMP2 are secreted by the cells and are neither influenced by TNF-alpha treatment nor by the origin of the muscular type (Fig. 2A–D). The secreted factors GRO, IL-6, MCP1, MCP3 and RANTES were all up-regulated by the TNF-alpha treatment without any difference between cells (Fig. 3A–F). RANTES secretion was the most sensitive to TNF-alpha treatment with a 27 fold increase in MC-T cells treated with TNF-alpha compared to control (Fig. 3F). Two factors, Angiogenin (ANG) and osteoprotegerin (OPG) were more secreted by MC-T than by MC-S cells (Fig. 3G,H). These data were supported by our RNA-seq data where TNFRSF11B (OPG) and ANG genes were more expressed in MC-T compared to MC-S (mean fold change = 4.88 ± 0.6 and 1.96 ± 0.11 respectively).

### Impacts of CM from different muscle origin and insulin sensitivity reveal a different impact on beta-cell function and survival

To study the impact of myokines secreted by different muscle cell types on beta-cells, we treated rat sorted beta-cells and human islets with conditioned medium from control and TNF-alpha treated MC-S and MC-T primary cultures. In order to compare the impact of myokines secreted by control and insulin-resistant cells, and not the impact of TNF-alpha by itself, TNF-alpha was added to the control CM after collection.

**Figure 4:**
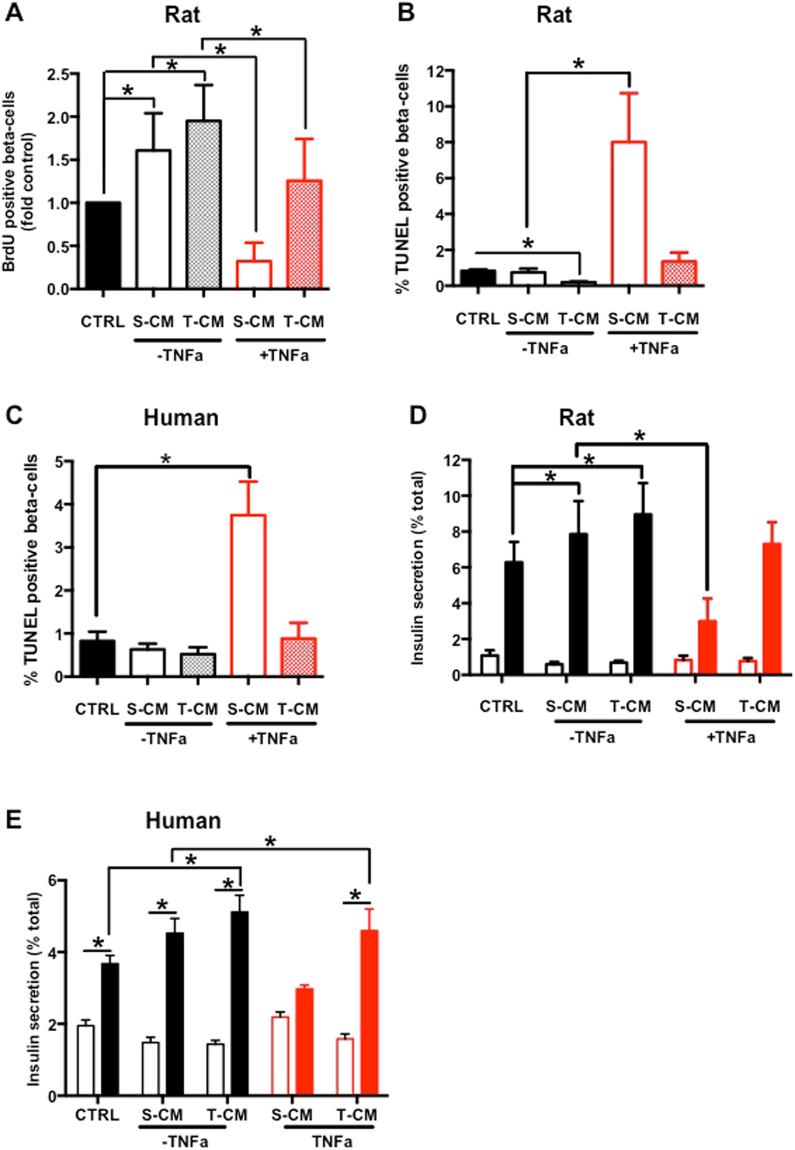
Impacts of CM from diverse muscle origin and insulin sensitivity reveal a different impact on beta-cell function and survival. Human islets, human dispersed islets or sorted rat beta-cells were cultured for 24 h with different CM. (**A**) Rat beta-cell proliferation. BrdU-positive beta-cells in sorted rat beta-cells; n = 9. (**B**) TUNEL-positive beta-cells in sorted rat beta-cells; n = 9. (**C**) TUNEL-positive beta-cells among dispersed human islet; n = 9. (**D**) Rat sorted beta-cells insulin secretion: 2.8 mM glucose (open bars), 16.7 mM glucose (closed bars); n = 9. (**E**) Human islet insulin secretion: 2.8 mM glucose (open bars), 16.7 mM glucose (closed bars); n = 9. *p < 0.05 for the indicated comparison as tested by ANOVA followed by Bonferroni post hoc test.

C-S-CM and C-T-CM both increased rat beta-cell proliferation with a higher impact of C-T-CM while TNF-alpha alone had no impact (data not shown). The CM of TNF-alpha treated myotubes had a different impact. TNF-S-CM strongly reduced rat beta-cell proliferation while TNF-T-CM had no significant impact on rat beta-cell proliferation compared to control condition (Fig. 4A).

C–S-CM did not change rat and human beta-cell apoptosis (Fig. 4B,C). C-T-CM reduced apoptosis in rat beta-cells and had no significant impact on human beta-cells. TNF-alpha did not impact apoptosis neither of rat beta-cells nor of human as previously reported^6^. TNF-S-CM strongly increased rat and human beta-cell apoptosis, whereas TNF-T-CM did not change rat nor human beta-cell apoptosis when compared to control condition. Note here that human beta-cell proliferation was not assessed as we have previously shown that human beta-cells have no replication rate *in vitro*^7^.

GSIS was increased after treatment with C-S-CM in rat sorted beta-cells and unchanged in human islets (Fig. 4D,E). The treatment with C-T-CM increased GSIS in both sorted rat beta-cells and human islets (8.95% and 5.11% of insulin content respectively). TNF-S-CM decrease GSIS in sorted rat beta-cells and human islets similarly to TNF-alpha action alone. Interestingly, TNF-T-CM had no negative impact on GSIS in both rat sorted beta-cells and human islets. Moreover, C-T-CM protected beta-cells from direct TNF-alpha impact on GSIS. Basal insulin secretion tends to decrease when sorted rat beta-cells and human islets were treated with C-S-CM and C-T-CM (Fig. 4D,E).

Here we have identified a positive impact of C-T-CM and TNF-T-CM on rat sorted beta-cells and human islets. We have hypothesized that this positive impact could be mediated by myokines secreted predominantly by MC-T. Considering the relative MC-T dependent secretion of ANG and OPG in the CM, we hypothesized that these two factors could mediate in part the protective impact of MC-T conditioned media on beta-cells function and survival. To test this hypothesis, rat sorted beta-cells were incubated with proinflammatory cytokines and/or with TNF-S-CM with different concentrations of ANG or OPG.

### ANG treatment counteracts cytomix and TNF-MC-S-CM impact on primary sorted rat beta-cells proliferation

**Figure 5:**
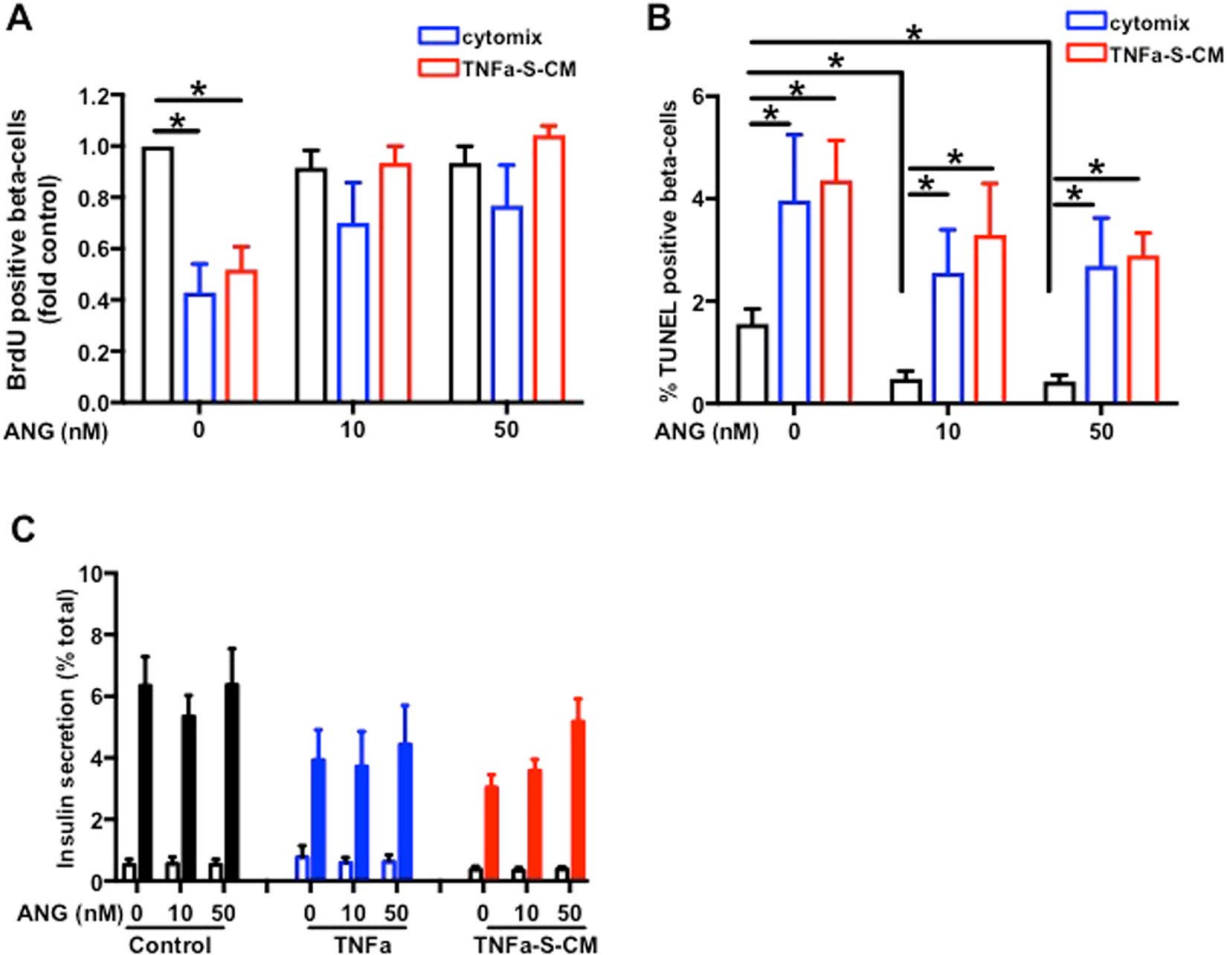
ANG treatment counteracts cytomix and TNF-SKMC-S-CM impact on primary sorted rat beta-cells proliferation. Rat sorted beta-cells were cultured for 24 h with different concentrations of ANG alone or in the presence of cytomix or of different CM. (**A**) Rat beta-cell proliferation. BrdU-positive beta-cells in sorted rat beta-cells; n = 14. (**B**) Beta-cell death. TUNEL-positive beta-cells in sorted rat beta-cells; n = 14. (**C**) Rat sorted beta-cells insulin secretion: 2.8 mM glucose (open bars), 16.7 mM glucose (closed bars); n = 14. *p < 0.05 for the indicated comparison by ANOVA followed by Bonferroni post hoc test.

At 10 and 50 nM, ANG alone did not impact beta-cells proliferation. Sorted rat beta-cells were exposed to TNF-alpha in combination with IL-1beta and IFN-gamma (cytomix), known to be cytotoxic towards beta-cells and believed to be implicated in decreased beta-cell function and mass in diabetic state. A 48 h treatment with cytomix (TNF-alpha, IL-1beta and INF-gamma, 20 ng/ml each) or TNF-S-CM reduced BrdU incorporation. The anti-proliferative effects of cytomix and TNF-S-CM were counteracted by 10 or 50 nM ANG (Fig. 5A).

ANG treatment at 10 and 50 nM alone decreased rat beta-cell apoptosis. Apoptosis was increased by cytomix and TNF-S-CM treatment. ANG was unable to prevent the apoptosis induced by cytomix or TNF-S-CM treatment (Fig. 5B).

GSIS was reduced by 24 h treatment with TNF**-**alpha or with TNF-S-CM. ANG alone had no impact on GSIS and was unable to preserve beta-cells GSIS impaired by 24 h treatment with TNF**-**alpha. However, ANG could partially preserve the insulin secretion impaired by 24 h treatment with TNF-S-CM (Fig. 5C).

### OPG protects beta-cells from TNF-alpha, cytomix and TNF-S-CM negative effects

**Figure 6:**
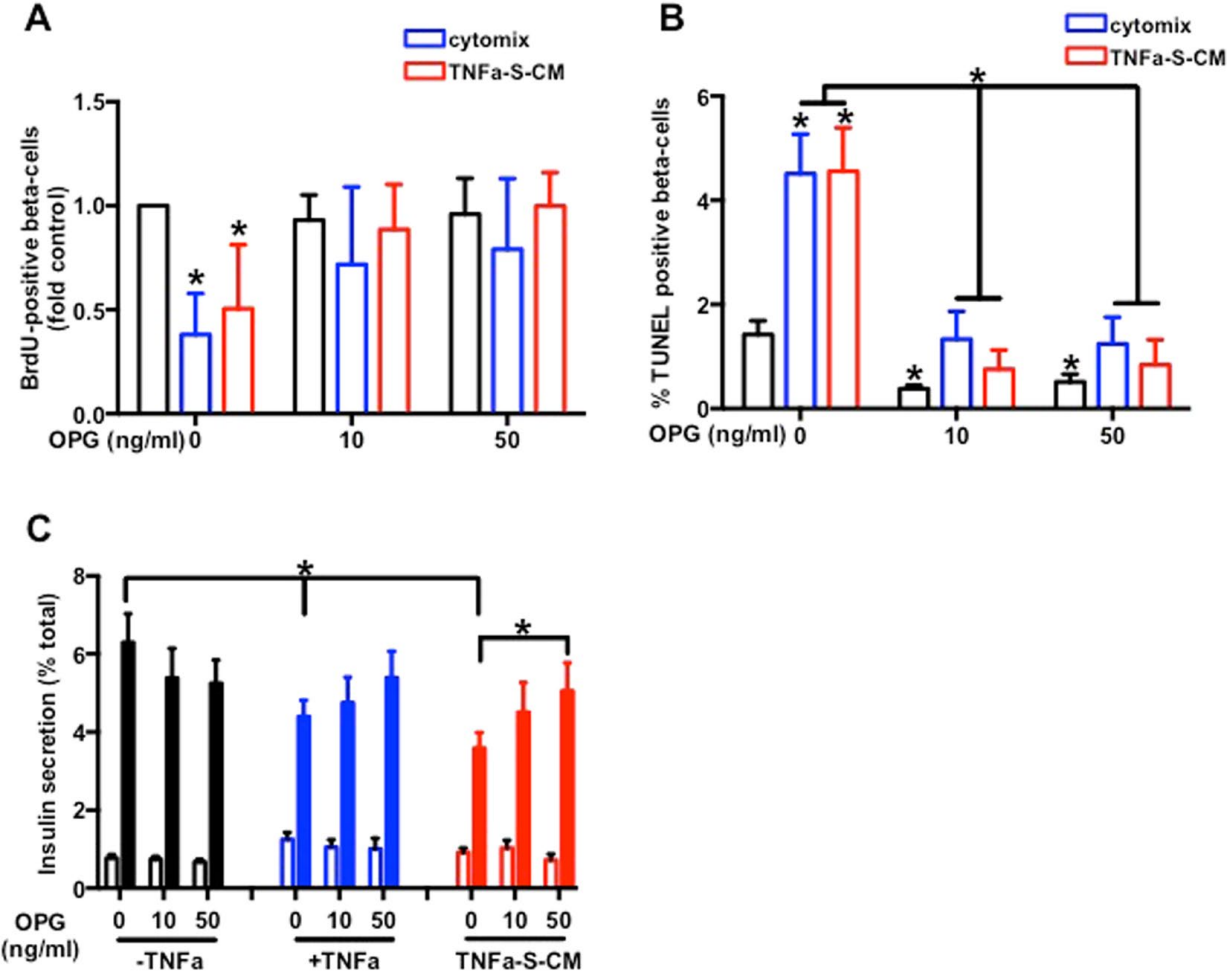
OPG protects beta-cells from TNF-alpha, cytomix and TNF-SKMC-S-CM negative effects. Rat sorted beta-cells were cultured for 24 h with different concentrations of OPG alone or in the presence of cytomix or of different CM. (**A**) Rat beta-cell proliferation. BrdU-positive beta-cells in sorted rat beta-cells; n = 12. (**B**) Beta-cell death. TUNEL-positive beta-cells in sorted rat beta-cells; n = 12. (**C**) Rat sorted beta-cells insulin secretion: 2.8 mM glucose (open bars), 16.7 mM glucose (closed bars); n = 12. *p < 0.05 for the indicated comparison as tested by ANOVA followed by Bonferroni post hoc test.

OPG alone did not impact sorted rat beta-cell proliferation, however it suppressed cytomix negative effect on proliferation at 10 and 50 ng/ml. TNF-S-CM induced decrease of rat sorted beta-cell proliferation was suppressed by 10 and 50 ng/ml OPG (Fig. 6A). OPG at 10 and 50 ng/ml reduced beta-cell apoptosis. OPG at 10 and 50 ng/ml totally suppressed cytomix and TNF-S-CM apoptosis induction (Fig. 6B). OPG alone had no impact on GSIS at 10 and 50 ng/ml and failed to protect beta-cells from the negative impact of TNF-alpha while it protects them from the adverse effect of TNF-S-CM (Fig. 6C).

## Discussion

In the present study, we have established human *in vitro* models of type I and type II muscles and showed that differentiated myotubes isolated from soleus or triceps skeletal biopsies present a unique signature with regards to gene expression and myokine secretion. Among the top differentially expressed genes between MC-S and MC-T, we found that EBF2 is up regulated in MC-T. Interestingly, this gene is known to be an important mitochondrial factor^8^. Furthermore, myonectin (encoded by FAM132B gene) known to induce free fatty acid metabolism^9^ was increased in MC-S compared to MC-T. Additionally, we have defined MC-S and MC-T specific myokine secretion profiles in basal condition and after TNF-alpha treatment known to induce insulin resistance^5^. Among all detected myokines, ANG and OPG are specific to MC-T. We do not exclude other possible myokine secretions to be muscle cell type dependent as we have used here a targeted approach to define different myokines groups. Surprisingly, MC-T were resistant to TNF-alpha induced insulin resistance, while MC-S developed insulin resistance under similar condition. We and others have demonstrated that silencing MAP4K4 in adipose tissue^10,11^, in beta-cells^6^ or in human skeletal muscle cells^5^ prevent TNF-alpha induced insulin resistance. While the expression of TNF-alpha receptor is similar in the two type of muscle cells, MAP4K4 expression is higher in MC-S compared to MC-T which can in part explain TNF-alpha resistance observed in MC-T. However, the precise mechanism underlying the TNF-alpha resistance of MC-T is currently investigated in another study. Our previous finding that TNF-alpha inhibits insulin-stimulated peripheral glucose uptake^12^ was based on studies over leg muscle, while it is not known if TNF-alpha has similar effects on arm muscle.

While CM from insulin sensitive MC-S/T exerted a beneficial effect on rat and human beta-cells, only TNF-S-CM exerted detrimental effects with a decrease in GSIS and rat beta-cells proliferation and an increase in apoptosis. Interestingly, C-T-CM protected beta-cells from TNF-alpha negative impact on GSIS and TNF-T-CM failed to mimic the impact of TNF-S-CM on sorted rat beta-cells and human islets. ANG and OPG mRNA expression and protein secretion were both increased in MC-T and MC-T-CM under basal condition and after TNF-alpha stimulation compare to MC-S and MC-S-CM. ANG treatment only reversed the negative impact of cytomix and TNF-S-CM on rat beta-cell proliferation without any impact on GSIS and apoptosis. Thought ANG did not reproduce C/TNF-CM impact on beta-cells, it remains an interesting myokine that promote beta-cell replication during diabetes etiology. OPG could also partly mediate the protective effect of C/TNF-T-CM as OPG treatment protects sorted rat beta-cells from TNF-alpha impact on GSIS, as well as cytomix and TNF-S-CM effects on proliferation, apoptosis and GSIS. Our results are supported by a study showing that IL-1beta effects on beta-cells were prevented by OPG pretreatment in an insulin secreting cell line^13^. Moreover, a recent study performed in rodents has shown that OPG injection can increase mouse beta-cells proliferation and delay hyperglycemia in diabetic mice by modulation of CREB and GSK3 pathway^14^.

In conclusion, we have demonstrated that MC-S and MC-T reveal distinct signatures with different profiles of myokine secretion in both basal state and after TNF-alpha treatment. We found that TNF-S-CM negatively impact human islets and rat sorted beta-cells function and survival while TNF-T-CM not. We have hypothesized that these phenomena could be mediated by triceps specific myokine(s). We have identified OPG as a benefic myokine, which protects beta-cells from TNF-alpha, cytomix and TNF-S-CM negative impacts. Therefore we believe that OPG may represent a potential treatment to improve beta-cell function in type I and type II diabetes.

## Methods

### Human primary muscle cells culture

Biopsies were obtained from healthy donors (Supplementary Table 3). The study participants have given their informed consent. The samples collection was performed in accordance with the Declaration of Helsinki and approved by the Ethical Committee of Copenhagen and Frederiksberg Communities, Denmark (H-3-2012-090). All methods were performed in accordance with the relevant guidelines and regulations.

Soleus (S) and triceps brachii (T) biopsies were collected using the percutaneous needle biopsy method. Primary human skeletal muscle cells (MC) were obtained as previously described in details^15^. To prepare conditioned medium (CM), differentiated myotubes where cultured overnight in DMEM without serum and then treated with 0 or 20 ng/ml TNF-alpha in DMEM without serum for 24 h.

### RNA-sequencing

We collected RNA from muscle biopsies (Cells) and differentiated myotubes^16^ prepared from S and T skeletal muscles. RNA extraction was performed on 14 samples per type of muscle for HM and 5 + 2 technical replicates for cells following standard RNeasy protocol (RNeasy, Qiagen). RNA-seq libraries were prepared with the Illumina TruSeq protocol following manufacturer’s instructions. 49 bp paired-ends reads were generated with an Illumina HiSeq2000 sequencer. Reads were mapped with GEMtools v1.6.2 (doi: 10.1038/nmeth.2221; http://gemtools.github.io/) onto the human genome GRCh37 and the gene annotation GENCODE 19. Reads having a proper orientation and a GEM mapping quality score of 150 or above were kept for the gene quantification step (read count per gene). These counts were used to generate read per kilobase per million reads (RPKM) as previously described^17^.

### Glucose uptake

Glucose uptake was measured as previously described in details^15^.

### Cytokine arrays

The presence of 119 different cytokines in CM was tested using Human Cytokine Antibody Array G-Series 6 and 7 (Ray Biotech, Norcross, GA) following manufacturer’s instructions.

### Human islets

Human islets were kindly provided by the Cell Isolation and Transplant Centre of the University of Geneva (JDRF award 31-2008-413, ECIT Islet for Basic Research Program). Human islets were dispersed by Acutase (PAA Laboratories, Austria). For functional analysis and immunostaining, human islets or non-sorted single cells were cultured in CMRL-1066 medium on plastic dishes coated with extracellular matrix secreted by 804 G rat bladder cancer cells (804 G ECM), as described elsewhere^18^ and were left in islet medium for 24 h to adhere and spread before initiation of the experiments.

### Rat sorted-beta cells isolation and culture

The protocol of rat islet isolation was approved by the State Commissioner on Animal Care (31.1.1061/2329/0). Wistar rats (150–200 g) pancreases were digested with collagenase and islets of Langerhans isolated using a Ficoll gradient. Islets were then digested with trypsin and the cells obtained sorted by FACS to separate the beta-cell and the non-beta-cell fractions. After sorting, 10000 or 30000 cells/well rat beta-cells were plated in 35 mm tissue culture dishes on 804 G matrix in DMEM supplemented with 10% Fetal Calf Serum and antibiotics^18^.

### Human islets and Rat sorted-beta cells treatments

Cells were treated for 24 h with or without TNF-alpha (20 ng/ml) alone or in combination with IL-1beta (20 ng/ml) and IFNgamma (20 ng/ml) or MC-S-CM or MC-T-CM. Cells were also incubated for 24 h with angiogenin (10 or 50 nM) or osteoprotegerin (10 or 50 ng/ml). Human TNF-alpha, human INF-gamma, rat IL-1beta, human Angiogenin and human osteoprotegerin where obtained from R&D Systems Europe Ltd (Abingdon, UK).

### Glucose Stimulated Insulin Secretion test (GSIS)

For acute insulin release in response to glucose, islets and beta-cells were preincubated for 2 h (2.8 mM glucose) and incubated in Krebs Ringer bicarbonate Hepes buffer, 0.5% BSA (KRB) containing 2.8 mM glucose for 1 h followed by 1 h incubation in KRB containing 16.7 mM glucose. Total insulin was extracted with 0.18 M HCl in 70% ethanol for determination of insulin content. Insulin was measured by radioimmunoassay.

### Immunofluorescence staining

Dispersed islet cells or sorted beta-cells were treated for 24 h in the presence of BrdU for proliferation measurement. The incorporated BrdU was detected with the BrdU-detection kit (Roche, Switzerland) and cells co-stained with anti-insulin antibody (Dako, Denmark). The free 3-OH strand breaks were detected by the terminal deoxynucleotidyl-transferase-mediated deoxyuridine 5-triphosphate nick-end labeling (TUNEL) technique according to the manufacturer’s instructions (*In situ* cell death detection kit, Roche, Switzerland). The numbers of TUNEL-positive or BrdU-positive beta-cells were determined by blind counting with a fluorescent microscope. For each condition in each individual experiment over 1000 cells were analyzed.

### Statistical analyses

Data are expressed as means ± SEM, with the number of individual experiments presented in the figure legends. All data were tested for normality and analyzed with PRISM (GraphPad, San Diego, CA). Differences were evaluated using Student’s t test or ANOVA with Bonferroni post hoc test for multiple comparison analysis. Significance was set as p < 0.05.

The differential gene expression analysis was performed with DESeq2^19^ starting with gene read counts and using the GC mean content of the sample libraries as covariate. Three types of comparisons were performed: between myotubes, between biopsies and between the same type of myotubes and biopsies. Genes expressed at 1 RPKM in at least 90% of the samples in either condition 1 or condition 2 were kept for the analysis. A gene was considered as differentially expressed if its adjusted p-value was <= 0.05 for HM vs. HM comparisons and if it was <= 0.05 in at least 80% of the technical replicate combinations for comparisons involving myotubes. Functional terms enriched for differentially expressed genes were assessed with WebGestalt^20^ using the differentially expressed genes as input and all tested genes as the reference set. Terms with an adjusted p-value <= 0.05 were reported (Supplementary Table 1). Hierarchical clustering and principal component analyses were performed on RPKM values using R.

## Electronic supplementary material

Supplementary information
