# Supplementary material for: Angiogenin and Osteoprotegerin are type II muscle specific myokines protecting pancreatic beta-cells against proinflammatory cytokines
Source: Sci Rep. 2018 Jul 3;8:10072. doi: 10.1038/s41598-018-28117-2 (PMC6030123; doi:10.1038/s41598-018-28117-2)
Supplement: Supplementary file 1 — Supplementary information [file 41598_2018_28117_MOESM1_ESM.pdf]

Angiogenin and Osteoprotegerin are type II muscle specific myokines protecting pancreatic beta-cells against proinflammatory cytokines

Sabine Rutti[1], Rodolphe Dusaulcy[2], Jakob S. Hansen[3,4] , Cédric Howald[5], Emmanouil T. Dermitzakis[5], Bente K. Pedersen[3], Michel Pinget[1] , Peter Plomgaard[3,4,*] and Karim Bouzakri[1, $,*]

1: UMR DIATHEC, EA 7294, Centre Européen d'Etude du Diabète, Université de Strasbourg, Fédération de Médecine Translationnelle de Strasbourg (FMTS), Bld René Leriche, 67200 Strasbourg, France..
2: Molecular Diabetes Laboratory, Division of Endocrinology-Diabetes-Hypertension and Nutrition, University Hospital/University of Geneva Medical School, 1211 Geneva, Switzerland.
3: Department of Clinical Biochemistry, Rigshospitalet, Copenhagen, Denmark.
4: Centre of Physical Activity Research, Rigshospitalet, Copenhagen, Denmark.
5: Department of Genetic Medicine and Development, University of Geneva Medical School 1211 Geneva, Switzerland; Institute for Genetics and Genomics in Geneva (iGE3), University of Geneva, 1211 Geneva, Switzerland; Swiss Institute of Bioinformatics, Geneva, Switzerland

* : Equally contribution

$ Correspondence:
Karim Bouzakri UMR DIATHEC, EA 7294, Centre Européen d'Etude du Diabète, Université de Strasbourg, Fédération de Médecine Translationnelle de Strasbourg (FMTS), Bld René Leriche, 67200 Strasbourg, France. Email : K.Bouzakri@Ceed-diabete.org

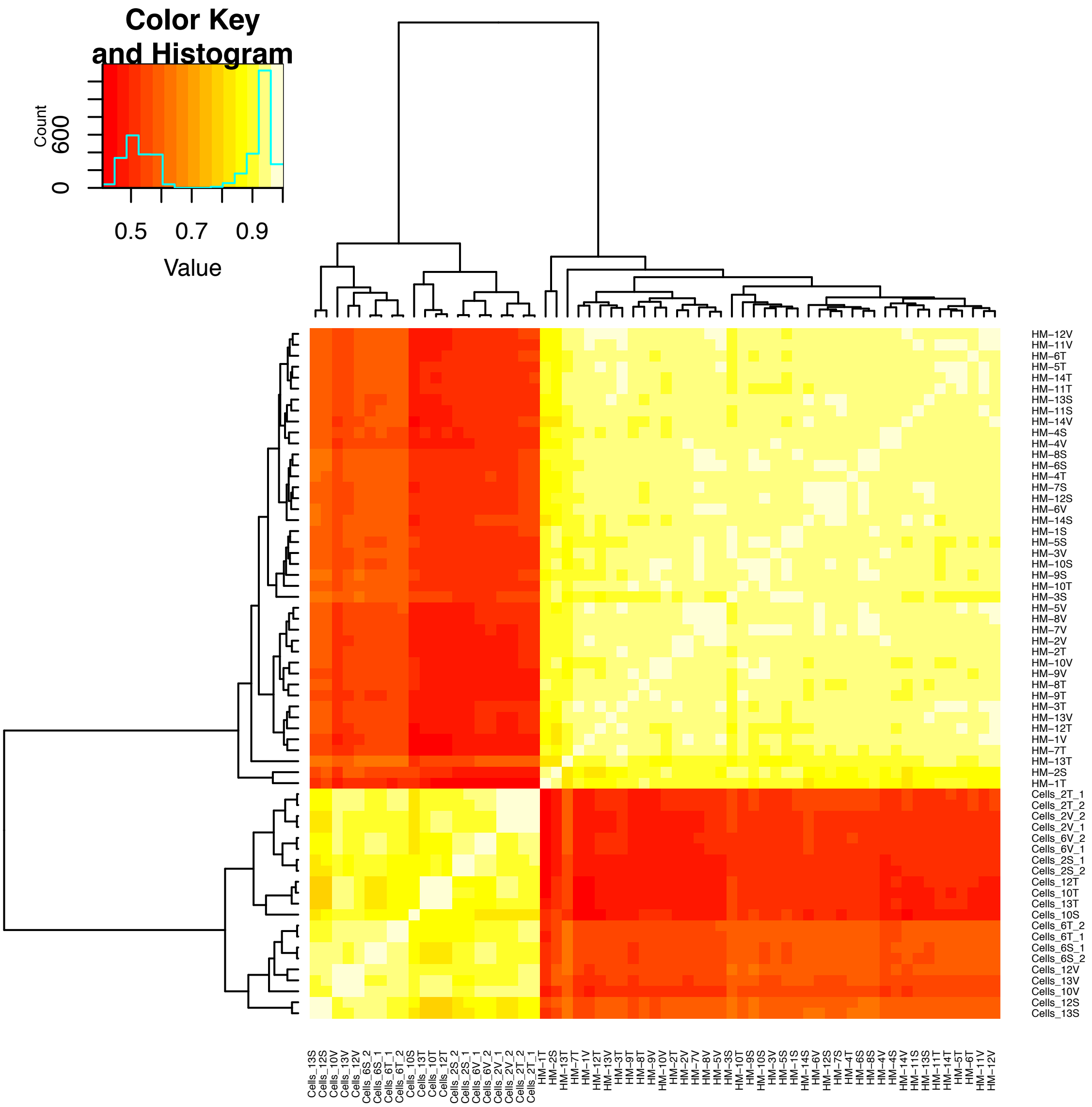

Figure S1: Hierarchical clustering of the spearman correlation coefficients of RPKM values. Genes having a RPKM value >= 1 in at least 90% of the samples were taken into account. Myotubes are well segregated from biopsies but the type of tissue (soleus, triceps and vastus) doesn't cluster together.

Figure S2

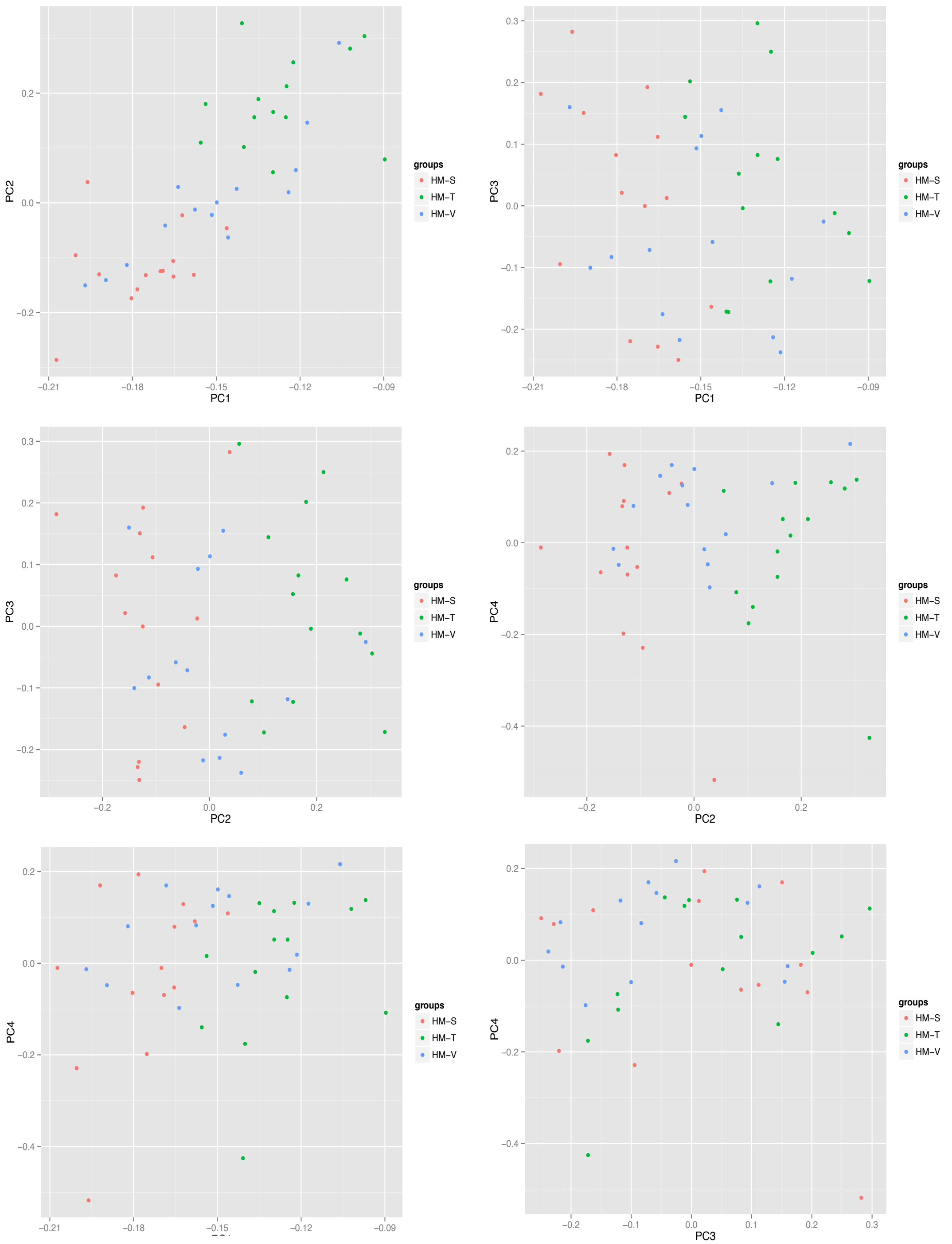

Figure S2: principal component analysis of the RPKM values of all genes with a RPKM >= 1 in at least 90% of the samples. Only biopsies were kept. Principal component 2 (PC2 axis) separates the soleus (HM-S) form the triceps (HM-T) whereas the vastus (HM-V) is spread all over PC2 suggesting the heterogeneous nature of this tissue (mix of type I and type II fibers).

Figure S3

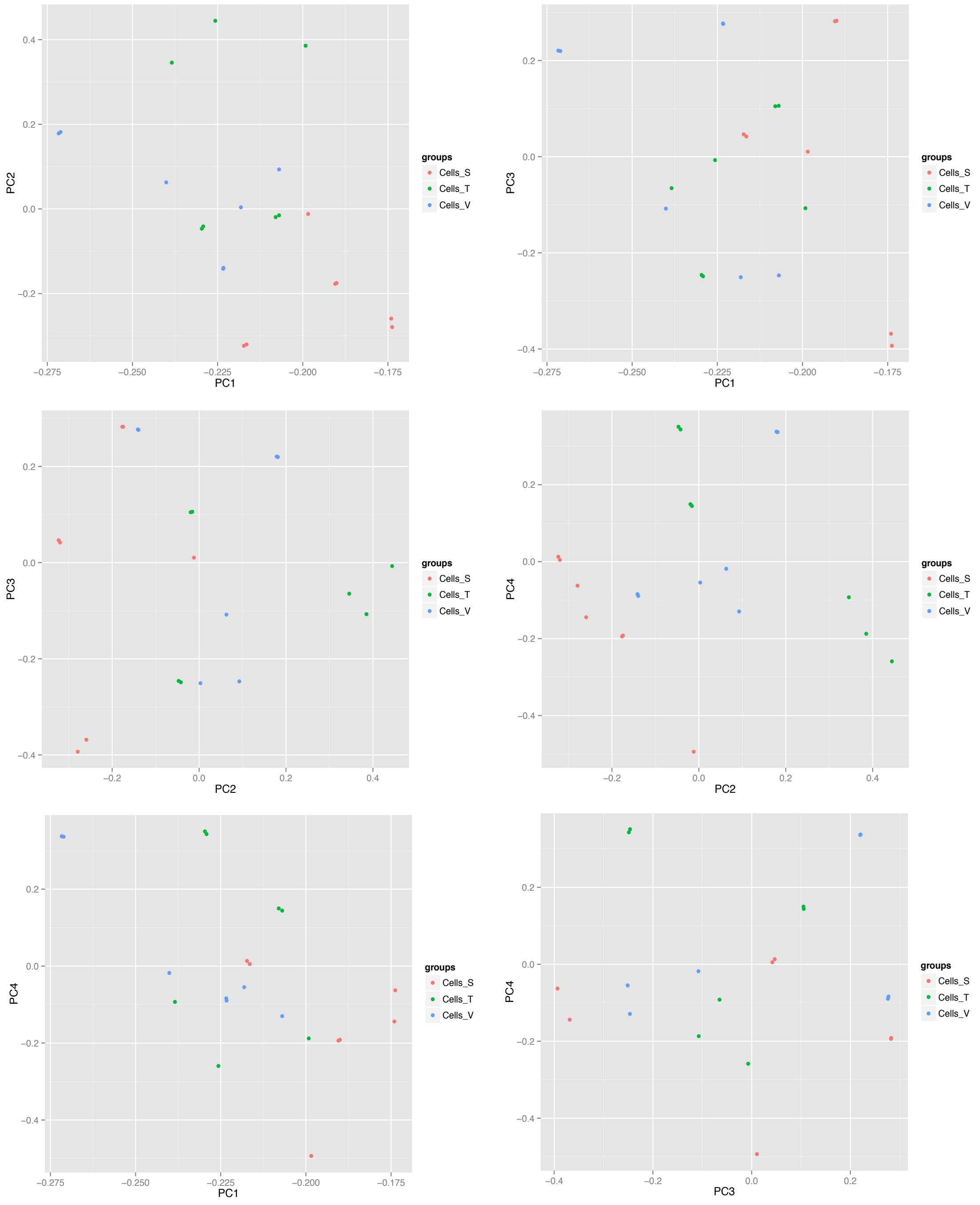

Figure S3: principal component analysis of the RPKM values of all genes with an RPKM >= 1 in at least 90% of the samples. Only myotubes were kept.

Table S1: GO terms and KEGG pathways of the the differentialy expressed genes in Cells-S versus Cells-T/Biopsies-S versus Biopsies-T (A and D), Cells-T versus Cells –S (B and E) and Biopsies-S versus Biopsies-T (C and F).

A **Cells-S_vs_Cells-T/Biopsies-S_vs_Biopsies-T, 864 genes**

GO terms	stats	adj_pvalue
organ_development	C=1538;O=167;E=112.45;R=1.49;rawP=1.54e-08	2.28E-05
system_development	C=2158;O=220;E=157.79;R=1.39;rawP=8.08e-09	2.28E-05
anatomical_structure_development	C=2485;O=244;E=181.70;R=1.34;rawP=2.67e-08	2.63E-05
developmental_process	C=2782;O=266;E=203.41;R=1.31;rawP=5.38e-08	3.98E-05
cell_migration	C=531;O=73;E=38.83;R=1.88;rawP=6.92e-08	4.09E-05
localization_of_cell	C=562;O=75;E=41.09;R=1.83;rawP=1.56e-07	6.59E-05
cell_motility	C=562;O=75;E=41.09;R=1.83;rawP=1.56e-07	6.59E-05
anatomical_structure_morphogenesis	C=1331;O=143;E=97.32;R=1.47;rawP=4.63e-07	1.00E-04
cellular_component_movement	C=754;O=92;E=55.13;R=1.67;rawP=3.84e-07	1.00E-04
multicellular_organismal_development	C=2475;O=238;E=180.96;R=1.32;rawP=2.99e-07	1.00E-04
locomotion	C=752;O=92;E=54.98;R=1.67;rawP=3.40e-07	1.00E-04
tissue_development	C=931;O=106;E=68.07;R=1.56;rawP=1.40e-06	3.00E-04
regulation_of_developmental_process	C=905;O=103;E=66.17;R=1.56;rawP=2.07e-06	5.00E-04
multicellular_organismal_process	C=3152;O=286;E=230.47;R=1.24;rawP=2.17e-06	5.00E-04
single-multicellular_organism_process	C=3142;O=284;E=229.73;R=1.24;rawP=3.51e-06	7.00E-04
skeletal_system_development	C=251;O=39;E=18.35;R=2.13;rawP=5.08e-06	9.00E-04
cell_differentiation	C=1568;O=157;E=114.65;R=1.37;rawP=8.45e-06	1.30E-03
immune_system_process	C=1012;O=110;E=73.99;R=1.49;rawP=7.90e-06	1.30E-03
cellular_developmental_process	C=1686;O=167;E=123.28;R=1.35;rawP=7.50e-06	1.30E-03
regulation_of_blood_vessel_endothelial_cell_migration	C=31;O=10;E=2.27;R=4.41;rawP=4.41e-05	6.50E-03
cell_fate_commitment	C=112;O=21;E=8.19;R=2.56;rawP=5.13e-05	7.20E-03
enzyme_linked_receptor_protein_signaling_pathway	C=628;O=72;E=45.92;R=1.57;rawP=6.67e-05	8.60E-03
single-organism_process	C=4462;O=374;E=326.25;R=1.15;rawP=6.95e-05	8.60E-03
actin_cytoskeleton_organization	C=310;O=42;E=22.67;R=1.85;rawP=6.68e-05	8.60E-03
biological_adhesion	C=533;O=62;E=38.97;R=1.59;rawP=0.0001	9.50E-03
skeletal_system_morphogenesis	C=118;O=21;E=8.63;R=2.43;rawP=0.0001	9.50E-03
blood_vessel_endothelial_cell_migration	C=48;O=12;E=3.51;R=3.42;rawP=0.0001	9.50E-03
regulation_of_cellular_response_to_growth_factor_stimulus	C=34;O=10;E=2.49;R=4.02;rawP=0.0001	9.50E-03
regionalization	C=174;O=27;E=12.72;R=2.12;rawP=0.0001	9.50E-03
cell_proliferation	C=990;O=103;E=72.39;R=1.42;rawP=9.87e-05	9.50E-03
cell_adhesion	C=532;O=62;E=38.90;R=1.59;rawP=0.0001	9.50E-03
negative_regulation_of_phosphorus_metabolic_process	C=188;O=28;E=13.75;R=2.04;rawP=0.0002	1.52E-02
metanephros_development	C=50;O=12;E=3.66;R=3.28;rawP=0.0002	1.52E-02
cell_surface_receptor_signaling_pathway	C=1364;O=133;E=99.73;R=1.33;rawP=0.0002	1.52E-02
organ_morphogenesis	C=487;O=57;E=35.61;R=1.60;rawP=0.0002	1.52E-02
connective_tissue_development	C=131;O=22;E=9.58;R=2.30;rawP=0.0002	1.52E-02
epithelium_development	C=357;O=45;E=26.10;R=1.72;rawP=0.0002	1.52E-02
negative_regulation_of_phosphate_metabolic_process	C=188;O=28;E=13.75;R=2.04;rawP=0.0002	1.52E-02
vasculature_development	C=368;O=46;E=26.91;R=1.71;rawP=0.0002	1.52E-02
peptide_cross-linking	C=14;O=6;E=1.02;R=5.86;rawP=0.0003	2.06E-02
growth_factor_binding	C=79;O=19;E=5.74;R=3.31;rawP=2.52e-06	1.40E-03
actin_binding	C=269;O=40;E=19.55;R=2.05;rawP=9.92e-06	2.80E-03
cytoskeletal_protein_binding	C=482;O=59;E=35.03;R=1.68;rawP=4.29e-05	8.10E-03
cell_adhesion_molecule_binding	C=35;O=10;E=2.54;R=3.93;rawP=0.0001	1.13E-02
calcium_ion_binding	C=350;O=45;E=25.44;R=1.77;rawP=0.0001	1.13E-02
extracellular_matrix_binding	C=30;O=9;E=2.18;R=4.13;rawP=0.0002	1.88E-02
glycosaminoglycan_binding	C=92;O=17;E=6.69;R=2.54;rawP=0.0003	2.42E-02
extracellular_region_part	C=482;O=70;E=35.37;R=1.98;rawP=1.79e-08	6.75E-06
cell_periphery	C=1999;O=205;E=146.68;R=1.40;rawP=4.59e-08	7.72E-06
plasma_membrane	C=1946;O=200;E=142.79;R=1.40;rawP=6.14e-08	7.72E-06
extracellular_region	C=815;O=100;E=59.80;R=1.67;rawP=1.14e-07	1.07E-05
plasma_membrane_part	C=889;O=102;E=65.23;R=1.56;rawP=2.15e-06	2.00E-04
extracellular_space	C=357;O=51;E=26.20;R=1.95;rawP=2.77e-06	2.00E-04
extracellular_matrix	C=226;O=37;E=16.58;R=2.23;rawP=2.85e-06	2.00E-04
stress_fiber	C=43;O=13;E=3.16;R=4.12;rawP=7.46e-06	4.00E-04
proteinaceous_extracellular_matrix	C=187;O=31;E=13.72;R=2.26;rawP=1.40e-05	5.00E-04
actin_filament_bundle	C=45;O=13;E=3.30;R=3.94;rawP=1.30e-05	5.00E-04
actomyosin	C=52;O=14;E=3.82;R=3.67;rawP=1.48e-05	5.00E-04
integral_to_plasma_membrane	C=496;O=62;E=36.39;R=1.70;rawP=2.00e-05	6.00E-04
intrinsic_to_plasma_membrane	C=519;O=64;E=38.08;R=1.68;rawP=2.26e-05	7.00E-04
cortical_cytoskeleton	C=37;O=11;E=2.71;R=4.05;rawP=4.49e-05	1.20E-03
adherens_junction	C=161;O=26;E=11.81;R=2.20;rawP=0.0001	2.50E-03
microvillus_membrane	C=9;O=5;E=0.66;R=7.57;rawP=0.0002	4.40E-03
microvillus	C=36;O=10;E=2.64;R=3.79;rawP=0.0002	4.40E-03
cell_cortex	C=134;O=22;E=9.83;R=2.24;rawP=0.0003	5.70E-03
anchoring_junction	C=170;O=26;E=12.47;R=2.08;rawP=0.0003	0.0057
cytoplasmic_vesicle_part	C=248;O=34;E=18.20;R=1.87;rawP=0.0003	0.0057
cell_cortex_part	C=61;O=13;E=4.48;R=2.90;rawP=0.0004	0.0072
Golgi_lumen	C=33;O=9;E=2.42;R=3.72;rawP=0.0005	0.0086
membrane	C=4169;O=348;E=305.90;R=1.14;rawP=0.0006	0.0094
cytoplasmic_membrane-bounded_vesicle_lumen	C=34;O=9;E=2.49;R=3.61;rawP=0.0006	0.0094
vesicle_lumen	C=35;O=9;E=2.57;R=3.50;rawP=0.0007	0.0106
neuronal_cell_body	C=156;O=23;E=11.45;R=2.01;rawP=0.0010	0.0145
lytic_vacuole	C=253;O=32;E=18.56;R=1.72;rawP=0.0017	0.0221
lysosome	C=253;O=32;E=18.56;R=1.72;rawP=0.0017	0.0221
cell_body	C=172;O=24;E=12.62;R=1.90;rawP=0.0017	0.0221
secretory_granule_lumen	C=33;O=8;E=2.42;R=3.30;rawP=0.0022	0.0266
cell_projection	C=717;O=73;E=52.61;R=1.39;rawP=0.0023	0.0266
platelet_alpha_granule	C=41;O=9;E=3.01;R=2.99;rawP=0.0024	0.0266
platelet_alpha_granule_lumen	C=33;O=8;E=2.42;R=3.30;rawP=0.0022	0.0266
cell_surface	C=248;O=31;E=18.20;R=1.70;rawP=0.0024	0.0266
cell-substrate_junction	C=119;O=18;E=8.73;R=2.06;rawP=0.0025	0.0269
lamellipodium	C=101;O=16;E=7.41;R=2.16;rawP=0.0027	0.0283
vesicle_membrane	C=220;O=28;E=16.14;R=1.73;rawP=0.0029	0.0295
apical_part_of_cell	C=141;O=20;E=10.35;R=1.93;rawP=0.0033	0.0327
cell-substrate_adherens_junction	C=114;O=17;E=8.36;R=2.03;rawP=0.0038	0.0367
cell_junction	C=427;O=46;E=31.33;R=1.47;rawP=0.0052	0.0461

B **Cells-S_vs_Cells-T, 2071 genes**

GO terms	stats	adj_pvalue
multicellular_organismal_process	C=2814;O=616;E=485.36;R=1.27;rawP=2.19e-15	5.90E-12
single-multicellular_organism_process	C=2806;O=614;E=483.98;R=1.27;rawP=2.84e-15	5.90E-12
developmental_process	C=2516;O=557;E=433.96;R=1.28;rawP=1.67e-14	2.31E-11
multicellular_organismal_development	C=2232;O=500;E=384.97;R=1.30;rawP=1.18e-13	1.23E-10
anatomical_structure_morphogenesis	C=1189;O=297;E=205.08;R=1.45;rawP=1.78e-13	1.48E-10
single-organism_process	C=4040;O=820;E=696.82;R=1.18;rawP=7.17e-13	4.97E-10
system_development	C=1935;O=439;E=333.75;R=1.32;rawP=1.22e-12	7.25E-10
anatomical_structure_development	C=2243;O=496;E=386.87;R=1.28;rawP=1.87e-12	9.72E-10
cellular_developmental_process	C=1514;O=352;E=261.13;R=1.35;rawP=2.27e-11	1.05E-08
anatomical_structure_formation_involved_in_morphogenesis	C=928;O=235;E=160.06;R=1.47;rawP=2.68e-11	1.11E-08
cell_differentiation	C=1400;O=326;E=241.47;R=1.35;rawP=1.39e-10	5.25E-08
organ_morphogenesis	C=436;O=126;E=75.20;R=1.68;rawP=4.16e-10	1.44E-07
nervous_system_development	C=954;O=235;E=164.55;R=1.43;rawP=4.88e-10	1.56E-07
cell_development	C=820;O=207;E=141.43;R=1.46;rawP=7.01e-10	2.08E-07
organ_development	C=1380;O=318;E=238.02;R=1.34;rawP=9.31e-10	2.58E-07
regulation_of_developmental_process	C=810;O=203;E=139.71;R=1.45;rawP=2.04e-09	5.30E-07
response_to_wounding	C=570;O=152;E=98.31;R=1.55;rawP=3.48e-09	8.51E-07
cellular_component_morphogenesis	C=598;O=157;E=103.14;R=1.52;rawP=6.15e-09	1.40E-06
extracellular_matrix_organization	C=123;O=48;E=21.21;R=2.26;rawP=6.71e-09	1.40E-06
extracellular_structure_organization	C=123;O=48;E=21.21;R=2.26;rawP=6.71e-09	1.40E-06
cell_morphogenesis	C=567;O=150;E=97.80;R=1.53;rawP=8.01e-09	1.59E-06
cell_proliferation	C=913;O=221;E=157.47;R=1.40;rawP=9.25e-09	1.75E-06
tissue_development	C=833;O=204;E=143.68;R=1.42;rawP=1.43e-08	2.59E-06
regulation_of_cell_differentiation	C=562;O=146;E=96.93;R=1.51;rawP=4.59e-08	7.95E-06
regulation_of_cell_proliferation	C=688;O=172;E=118.67;R=1.45;rawP=5.05e-08	8.40E-06
positive_regulation_of_cell_differentiation	C=274;O=82;E=47.26;R=1.74;rawP=1.04e-07	1.66E-05
positive_regulation_of_developmental_process	C=373;O=104;E=64.33;R=1.62;rawP=1.16e-07	1.79E-05
locomotion	C=674;O=167;E=116.25;R=1.44;rawP=1.55e-07	2.30E-05
localization_of_cell	C=508;O=132;E=87.62;R=1.51;rawP=2.11e-07	2.74E-05
cell_motility	C=508;O=132;E=87.62;R=1.51;rawP=2.11e-07	2.74E-05
regulation_of_biological_quality	C=1342;O=298;E=231.47;R=1.29;rawP=1.99e-07	2.74E-05
cellular_component_movement	C=676;O=167;E=116.60;R=1.43;rawP=1.91e-07	2.74E-05
regulation_of_multicellular_organismal_development	C=600;O=151;E=103.49;R=1.46;rawP=2.34e-07	2.95E-05
regulation_of_localization	C=733;O=178;E=126.43;R=1.41;rawP=2.56e-07	2.97E-05
skeletal_system_development	C=227;O=70;E=39.15;R=1.79;rawP=2.57e-07	2.97E-05
positive_regulation_of_cell_proliferation	C=351;O=98;E=60.54;R=1.62;rawP=2.53e-07	2.97E-05
regulation_of_multicellular_organismal_process	C=914;O=214;E=157.65;R=1.36;rawP=2.81e-07	3.16E-05
cell_migration	C=478;O=125;E=82.45;R=1.52;rawP=3.12e-07	3.41E-05
cell_adhesion	C=484;O=126;E=83.48;R=1.51;rawP=3.64e-07	3.88E-05
biological_adhesion	C=485;O=126;E=83.65;R=1.51;rawP=4.11e-07	4.27E-05
transmembrane_signaling_receptor_activity	C=204;O=63;E=35.11;R=1.79;rawP=8.98e-07	3.00E-04
signal_transducer_activity	C=458;O=118;E=78.83;R=1.50;rawP=1.39e-06	3.00E-04
molecular_transducer_activity	C=458;O=118;E=78.83;R=1.50;rawP=1.39e-06	3.00E-04
receptor_activity	C=343;O=95;E=59.03;R=1.61;rawP=5.26e-07	3.00E-04
signaling_receptor_activity	C=248;O=69;E=42.68;R=1.62;rawP=1.63e-05	2.60E-03
G-protein_coupled_receptor_activity	C=69;O=26;E=11.88;R=2.19;rawP=3.84e-05	5.20E-03
receptor_binding	C=600;O=136;E=103.27;R=1.32;rawP=0.0002	2.31E-02
amino_acid_transmembrane_transporter_activity	C=35;O=15;E=6.02;R=2.49;rawP=0.0003	2.43E-02
protease_binding	C=35;O=15;E=6.02;R=2.49;rawP=0.0003	2.43E-02
3',5'-cyclic-nucleotide_phosphodiesterase_activity	C=10;O=7;E=1.72;R=4.07;rawP=0.0003	2.43E-02
lipid_binding	C=408;O=95;E=70.22;R=1.35;rawP=0.0008	4.98E-02
neutral_amino_acid_transmembrane_transporter_activity	C=14;O=8;E=2.41;R=3.32;rawP=0.0008	4.98E-02
cyclic-nucleotide_phosphodiesterase_activity	C=11;O=7;E=1.89;R=3.70;rawP=0.0008	4.98E-02
extracellular_region	C=707;O=218;E=121.89;R=1.79;rawP=1.46e-20	6.88E-18
extracellular_region_part	C=416;O=128;E=71.72;R=1.78;rawP=2.93e-12	6.90E-10
proteinaceous_extracellular_matrix	C=164;O=62;E=28.27;R=2.19;rawP=2.02e-10	3.17E-08
intrinsic_to_membrane	C=2210;O=477;E=381.01;R=1.25;rawP=7.28e-10	8.57E-08
cell_periphery	C=1771;O=391;E=305.32;R=1.28;rawP=2.94e-09	2.77E-07
extracellular_matrix	C=199;O=68;E=34.31;R=1.98;rawP=4.13e-09	3.24E-07
plasma_membrane	C=1722;O=380;E=296.88;R=1.28;rawP=5.80e-09	3.90E-07
integral_to_membrane	C=2176;O=464;E=375.15;R=1.24;rawP=8.90e-09	4.66E-07
extracellular_space	C=303;O=92;E=52.24;R=1.76;rawP=8.04e-09	4.66E-07
extracellular_matrix_part	C=92;O=38;E=15.86;R=2.40;rawP=4.25e-08	2.00E-06
plasma_membrane_part	C=759;O=185;E=130.85;R=1.41;rawP=1.19e-07	5.10E-06
membrane	C=3791;O=743;E=653.58;R=1.14;rawP=3.47e-07	1.36E-05
intrinsic_to_plasma_membrane	C=434;O=115;E=74.82;R=1.54;rawP=4.81e-07	1.74E-05
membrane_part	C=2697;O=546;E=464.97;R=1.17;rawP=6.98e-07	2.35E-05
integral_to_plasma_membrane	C=415;O=108;E=71.55;R=1.51;rawP=2.72e-06	8.54E-05
collagen	C=37;O=16;E=6.38;R=2.51;rawP=0.0002	0.0059
membrane_raft	C=120;O=36;E=20.69;R=1.74;rawP=0.0004	0.0111
endoplasmic_reticulum_lumen	C=106;O=32;E=18.27;R=1.75;rawP=0.0007	0.0183
cell_projection	C=660;O=144;E=113.79;R=1.27;rawP=0.0010	0.0248
cell_junction	C=379;O=88;E=65.34;R=1.35;rawP=0.0015	0.0336
basement_membrane	C=51;O=18;E=8.79;R=2.05;rawP=0.0015	0.0336
axon	C=137;O=37;E=23.62;R=1.57;rawP=0.0026	0.049
caveola	C=45;O=16;E=7.76;R=2.06;rawP=0.0024	0.049
Golgi_stack	C=87;O=26;E=15.00;R=1.73;rawP=0.0025	0.049
microfibril	C=7;O=5;E=1.21;R=4.14;rawP=0.0023	0.049

C **Biopsies-S_vs_Biopsies-T, 3725 genes**

GO terms	stats	adj_pvalue
cellular_respiration	C=136;O=103;E=48.48;R=2.12;rawP=7.28e-22	3.65E-18
respiratory_electron_transport_chain	C=96;O=78;E=34.22;R=2.28;rawP=3.60e-20	9.02E-17
energy_derivation_by_oxidation_of_organic_compounds	C=233;O=147;E=83.06;R=1.77;rawP=4.65e-18	7.76E-15
generation_of_precursor_metabolites_and_energy	C=311;O=182;E=110.87;R=1.64;rawP=4.06e-17	5.08E-14
oxidation-reduction_process	C=370;O=208;E=131.90;R=1.58;rawP=1.02e-16	9.60E-14
electron_transport_chain	C=118;O=86;E=42.07;R=2.04;rawP=1.15e-16	9.60E-14
mitochondrial_ATP_synthesis_coupled_electron_transport	C=47;O=37;E=16.76;R=2.21;rawP=1.74e-09	1.09E-06
ATP_synthesis_coupled_electron_transport	C=47;O=37;E=16.76;R=2.21;rawP=1.74e-09	1.09E-06
acetyl-CoA_metabolic_process	C=41;O=33;E=14.62;R=2.26;rawP=4.81e-09	2.44E-06
oxidative_phosphorylation	C=57;O=42;E=20.32;R=2.07;rawP=4.88e-09	2.44E-06
homophilic_cell_adhesion	C=37;O=30;E=13.19;R=2.27;rawP=1.80e-08	8.20E-06
mitochondrial_electron_transport,_NADH_to_ubiquinone	C=36;O=29;E=12.83;R=2.26;rawP=4.13e-08	1.72E-05
aerobic_respiration	C=38;O=30;E=13.55;R=2.21;rawP=5.62e-08	2.17E-05
small_molecule_metabolic_process	C=1435;O=598;E=511.57;R=1.17;rawP=7.74e-08	2.77E-05
cofactor_catabolic_process	C=32;O=26;E=11.41;R=2.28;rawP=1.55e-07	5.18E-05
tricarboxylic_acid_cycle	C=24;O=21;E=8.56;R=2.45;rawP=2.18e-07	6.82E-05
muscle_system_process	C=182;O=98;E=64.88;R=1.51;rawP=2.93e-07	8.63E-05
catabolic_process	C=1301;O=542;E=463.80;R=1.17;rawP=4.22e-07	1.00E-04
striated_muscle_contraction	C=62;O=41;E=22.10;R=1.85;rawP=9.32e-07	2.00E-04
organic_substance_catabolic_process	C=1207;O=504;E=430.29;R=1.17;rawP=8.82e-07	2.00E-04
acetyl-CoA_catabolic_process	C=25;O=21;E=8.91;R=2.36;rawP=9.03e-07	2.00E-04
actin-myosin_filament_sliding	C=34;O=26;E=12.12;R=2.15;rawP=1.35e-06	2.00E-04
translational_termination	C=88;O=54;E=31.37;R=1.72;rawP=7.02e-07	2.00E-04
translational_elongation	C=104;O=61;E=37.08;R=1.65;rawP=1.25e-06	2.00E-04
organic_acid_catabolic_process	C=121;O=69;E=43.14;R=1.60;rawP=1.10e-06	2.00E-04
muscle_filament_sliding	C=34;O=26;E=12.12;R=2.15;rawP=1.35e-06	2.00E-04
carboxylic_acid_catabolic_process	C=121;O=69;E=43.14;R=1.60;rawP=1.10e-06	2.00E-04
muscle_contraction	C=156;O=85;E=55.61;R=1.53;rawP=9.29e-07	2.00E-04
coenzyme_catabolic_process	C=28;O=23;E=9.98;R=2.30;rawP=5.79e-07	2.00E-04
protein_complex_subunit_organization	C=567;O=255;E=202.13;R=1.26;rawP=1.19e-06	2.00E-04
skeletal_muscle_contraction	C=19;O=17;E=6.77;R=2.51;rawP=1.77e-06	3.00E-04
protein_complex_disassembly	C=122;O=69;E=43.49;R=1.59;rawP=1.65e-06	3.00E-04
cellular_protein_complex_disassembly	C=119;O=67;E=42.42;R=1.58;rawP=2.90e-06	4.00E-04
acyl-CoA_metabolic_process	C=64;O=41;E=22.82;R=1.80;rawP=3.21e-06	5.00E-04
thioester_metabolic_process	C=64;O=41;E=22.82;R=1.80;rawP=3.21e-06	5.00E-04
translational_initiation	C=140;O=76;E=49.91;R=1.52;rawP=4.19e-06	6.00E-04
cellular_catabolic_process	C=1145;O=473;E=408.18;R=1.16;rawP=8.92e-06	1.20E-03
single-organism_catabolic_process	C=149;O=79;E=53.12;R=1.49;rawP=8.92e-06	1.20E-03
small_molecule_catabolic_process	C=148;O=78;E=52.76;R=1.48;rawP=1.35e-05	1.70E-03
protein_targeting_to_membrane	C=133;O=71;E=47.41;R=1.50;rawP=1.85e-05	2.30E-03
oxidoreductase_activity	C=391;O=204;E=139.29;R=1.46;rawP=4.56e-12	5.34E-09
structural_constituent_of_ribosome	C=147;O=86;E=52.37;R=1.64;rawP=9.76e-09	5.71E-06
NADH_dehydrogenase_(quinone)_activity	C=37;O=29;E=13.18;R=2.20;rawP=1.24e-07	2.90E-05
NADH_dehydrogenase_activity	C=37;O=29;E=13.18;R=2.20;rawP=1.24e-07	2.90E-05
NADH_dehydrogenase_(ubiquinone)_activity	C=37;O=29;E=13.18;R=2.20;rawP=1.24e-07	2.90E-05
cofactor_binding	C=155;O=83;E=55.22;R=1.50;rawP=3.08e-06	5.00E-04
oxidoreductase_activity,_acting_on_NADH_or_NADPH,_quino	C=46;O=32;E=16.39;R=1.95;rawP=2.75e-06	5.00E-04
inorganic_cation_transmembrane_transporter_activity	C=171;O=90;E=60.92;R=1.48;rawP=3.13e-06	5.00E-04
ion_transmembrane_transporter_activity	C=284;O=137;E=101.17;R=1.35;rawP=5.78e-06	8.00E-04
hydrogen_ion_transmembrane_transporter_activity	C=70;O=43;E=24.94;R=1.72;rawP=8.97e-06	1.00E-03
structural_molecule_activity	C=357;O=165;E=127.18;R=1.30;rawP=1.56e-05	1.70E-03
transmembrane_transporter_activity	C=339;O=156;E=120.77;R=1.29;rawP=3.55e-05	2.80E-03
acyl-CoA_dehydrogenase_activity	C=13;O=12;E=4.63;R=2.59;rawP=3.59e-05	2.80E-03
coenzyme_binding	C=114;O=62;E=40.61;R=1.53;rawP=2.87e-05	0.0028
substrate-specific_transmembrane_transporter_activity	C=319;O=148;E=113.64;R=1.30;rawP=3.38e-05	0.0028
monovalent_inorganic_cation_transmembrane_transporter_a	C=115;O=62;E=40.97;R=1.51;rawP=4.09e-05	0.003
cation_transmembrane_transporter_activity	C=211;O=103;E=75.17;R=1.37;rawP=4.53e-05	0.0031
oxidoreductase_activity,_acting_on_the_CH-CH_group_of_dor	C=41;O=27;E=14.61;R=1.85;rawP=7.62e-05	0.005
oxidoreductase_activity,_acting_on_NADH_or_NADPH	C=65;O=38;E=23.16;R=1.64;rawP=0.0001	0.0062
NAD_binding	C=37;O=24;E=13.18;R=1.82;rawP=0.0003	0.0175
flavin_adenine_dinucleotide_binding	C=42;O=26;E=14.96;R=1.74;rawP=0.0005	0.0279
mitochondrial_part	C=586;O=322;E=207.58;R=1.55;rawP=9.02e-24	5.88E-21
mitochondrial_inner_membrane	C=278;O=178;E=98.47;R=1.81;rawP=5.23e-23	1.70E-20
organelle_inner_membrane	C=300;O=182;E=106.27;R=1.71;rawP=1.06e-19	1.73E-17
mitochondrial_membrane	C=388;O=223;E=137.44;R=1.62;rawP=9.37e-20	1.73E-17
mitochondrial_envelope	C=406;O=230;E=143.82;R=1.60;rawP=2.85e-19	3.72E-17
mitochondrion	C=1156;O=543;E=409.48;R=1.33;rawP=1.44e-18	1.56E-16
mitochondrial_membrane_part	C=121;O=84;E=42.86;R=1.96;rawP=1.88e-14	1.75E-12
mitochondrial_matrix	C=237;O=136;E=83.95;R=1.62;rawP=2.20e-12	1.79E-10
respiratory_chain	C=65;O=50;E=23.02;R=2.17;rawP=8.33e-12	6.03E-10
organelle_envelope	C=600;O=290;E=212.53;R=1.36;rawP=9.80e-12	6.39E-10
mitochondrial_respiratory_chain	C=60;O=47;E=21.25;R=2.21;rawP=1.12e-11	6.64E-10
envelope	C=601;O=290;E=212.89;R=1.36;rawP=1.25e-11	6.79E-10
mitochondrial_respiratory_chain_complex_I	C=39;O=32;E=13.81;R=2.32;rawP=2.81e-09	1.22E-07
respiratory_chain_complex_I	C=39;O=32;E=13.81;R=2.32;rawP=2.81e-09	1.22E-07
NADH_dehydrogenase_complex	C=39;O=32;E=13.81;R=2.32;rawP=2.81e-09	1.22E-07
ribosomal_subunit	C=128;O=76;E=45.34;R=1.68;rawP=2.27e-08	9.25E-07
ribosome	C=189;O=103;E=66.95;R=1.54;rawP=4.64e-08	1.78E-06
cytosolic_ribosome	C=86;O=53;E=30.46;R=1.74;rawP=5.90e-07	2.14E-05
cytosolic_part	C=158;O=84;E=55.97;R=1.50;rawP=3.09e-06	0.0001
contractile_fiber_part	C=120;O=67;E=42.51;R=1.58;rawP=3.39e-06	0.0001
cytoplasmic_part	C=4185;O=1573;E=1482.43;R=1.06;rawP=1.12e-05	0.0003
contractile_fiber	C=133;O=71;E=47.11;R=1.51;rawP=1.47e-05	0.0004
myofibril	C=124;O=67;E=43.92;R=1.53;rawP=1.51e-05	0.0004
striated_muscle_thin_filament	C=13;O=12;E=4.60;R=2.61;rawP=3.37e-05	0.0009
sarcomere	C=105;O=57;E=37.19;R=1.53;rawP=5.37e-05	0.0013
small_ribosomal_subunit	C=59;O=36;E=20.90;R=1.72;rawP=5.15e-05	0.0013
cytosolic_large_ribosomal_subunit	C=49;O=31;E=17.36;R=1.79;rawP=6.36e-05	0.0015
cytoplasm	C=5398;O=1988;E=1912.10;R=1.04;rawP=6.94e-05	0.0016
mitochondrial_proton-transporting_ATP_synthase_complex	C=19;O=15;E=6.73;R=2.23;rawP=0.0001	0.0022
large_ribosomal_subunit	C=70;O=40;E=24.80;R=1.61;rawP=0.0002	0.0042
sarcoplasm	C=49;O=30;E=17.36;R=1.73;rawP=0.0002	0.0042
proton-transporting_ATP_synthase_complex	C=20;O=15;E=7.08;R=2.12;rawP=0.0004	0.0082
membrane_part	C=2301;O=878;E=815.07;R=1.08;rawP=0.0006	0.0115
sarcoplasmic_reticulum	C=45;O=27;E=15.94;R=1.69;rawP=0.0006	0.0115
troponin_complex	C=7;O=7;E=2.48;R=2.82;rawP=0.0007	0.013
cytosolic_small_ribosomal_subunit	C=35;O=22;E=12.40;R=1.77;rawP=0.0008	0.0141
membrane	C=3251;O=1218;E=1151.58;R=1.06;rawP=0.0008	0.0141
ion_channel_complex	C=46;O=27;E=16.29;R=1.66;rawP=0.0010	0.0172
T-tubule	C=18;O=13;E=6.38;R=2.04;rawP=0.0016	0.0254
mitochondrial_nucleoid	C=32;O=20;E=11.34;R=1.76;rawP=0.0016	0.0254

D **Cells-S_vs_Cells-T/Biopsies-S_vs_Biopsies-T, 864 genes**

KEGG pathway	stats	adj_pvalue
Regulation_of_actin_cytoskeleton	C=154;O=26;E=10.55;R=2.46;rawP=1.56e-05	2.20E-03
Focal_adhesion	C=158;O=24;E=10.82;R=2.22;rawP=0.0002	1.06E-02
Pathogenic_Escherichia_coli_infection	C=40;O=10;E=2.74;R=3.65;rawP=0.0003	1.06E-02
Leukocyte_transendothelial_migration	C=71;O=14;E=4.86;R=2.88;rawP=0.0003	1.06E-02

E **Cells-S_vs_Cells-T, 2071 genes**

KEGG pathway	stats	adj_pvalue
Protein_digestion_and_absorption	C=32;O=17;E=5.37;R=3.17;rawP=2.71e-06	5.00E-04
Cytokine-cytokine_receptor_interaction	C=68;O=25;E=11.40;R=2.19;rawP=5.65e-05	5.50E-03
ECM-receptor_interaction	C=51;O=19;E=8.55;R=2.22;rawP=0.0004	0.0241
Focal_adhesion	C=143;O=40;E=23.98;R=1.67;rawP=0.0005	0.0241
Nitrogen_metabolism	C=9;O=6;E=1.51;R=3.98;rawP=0.0012	0.0386
Gap_junction	C=55;O=19;E=9.22;R=2.06;rawP=0.0010	0.0386
Amoebiasis	C=57;O=19;E=9.56;R=1.99;rawP=0.0017	0.041
Pathways_in_cancer	C=219;O=54;E=36.73;R=1.47;rawP=0.0017	0.041

F **Biopsies-S_vs_Biopsies-T, 3725 genes**

KEGG pathway	stats	adj_pvalue
Oxidative_phosphorylation	C=104;O=76;E=36.63;R=2.08;rawP=2.89e-15	6.10E-13
Alzheimer's_disease	C=131;O=89;E=46.13;R=1.93;rawP=1.73e-14	1.83E-12
Parkinson's_disease	C=102;O=73;E=35.92;R=2.03;rawP=6.18e-14	4.35E-12
Huntington's_disease	C=139;O=84;E=48.95;R=1.72;rawP=9.38e-10	4.95E-08
Cardiac_muscle_contraction	C=48;O=36;E=16.90;R=2.13;rawP=2.04e-08	8.61E-07
Valine,_leucine_and_isoleucine_degradation	C=37;O=29;E=13.03;R=2.23;rawP=9.44e-08	2.85E-06
Ribosome	C=85;O=54;E=29.93;R=1.80;rawP=8.84e-08	2.85E-06
Metabolic_pathways	C=645;O=287;E=227.15;R=1.26;rawP=2.85e-07	7.52E-06
Citrate_cycle_(TCA_cycle)	C=26;O=21;E=9.16;R=2.29;rawP=2.50e-06	5.86E-05
Calcium_signaling_pathway	C=66;O=41;E=23.24;R=1.76;rawP=7.18e-06	0.0002
Propanoate_metabolism	C=25;O=19;E=8.80;R=2.16;rawP=3.71e-05	0.0007
Fatty_acid_metabolism	C=31;O=22;E=10.92;R=2.02;rawP=5.29e-05	0.0009
Glycolysis_/_Gluconeogenesis	C=37;O=25;E=13.03;R=1.92;rawP=6.03e-05	0.001
Pyruvate_metabolism	C=30;O=19;E=10.57;R=1.80;rawP=0.0015	0.0226
Tight_junction	C=80;O=41;E=28.17;R=1.46;rawP=0.0023	0.0324

Table S2: Cytokine assay of the CM of SKMC-S and SKMC-T cells in control conditions of after 24h TNF-alpha treatment.

Cytokine	C-SKMC-S-CM		C-SKMC-T-CM		TNF-SKMC-S-CM		TNF-SKMC-T-CM	
	Protein level (fold C-SKMC-S-CM)	ESM (±)	Protein level (fold C-SKMC-S-CM)	ESM (±)	Protein level (fold C-SKMC-S-CM)	ESM (±)	Protein level (fold C-SKMC-S-CM)	ESM (±)
Angiogenin	1.00	0.22	4.92	1.54	1.30	0.28	3.17	1.05
Acrp30	1.00	0.31	1.06	0.16	1.02	0.12	1.20	0.13
AgRP	1.00	0.25	1.04	0.21	1.01	0.21	1.15	0.13
ANGPT2	1.00	0.23	1.00	0.18	1.06	0.17	1.21	0.07
AREG	1.00	0.22	0.97	0.22	0.98	0.22	0.99	0.18
Axl	1.00	0.23	1.03	0.17	0.98	0.19	1.10	0.11
b-NGF	1.00	0.17	1.03	0.17	1.05	0.35	1.13	0.12
BDNF	1.00	0.18	0.84	0.07	0.96	0.07	0.99	0.06
bFGF	1.00	0.26	1.00	0.15	1.02	0.20	1.15	0.09
BLC	1.00	0.15	0.90	0.07	1.04	0.07	1.08	0.12
BMP-4	1.00	0.12	0.97	0.05	1.09	0.09	1.19	0.11
BMP-6	1.00	0.16	0.89	0.14	1.13	0.08	1.16	0.06
BTC	1.00	0.27	1.03	0.21	0.98	0.14	1.21	0.12
CCL11	1.00	0.17	1.04	0.27	1.19	0.29	2.11	0.70
CCL24	1.00	0.12	0.89	0.09	1.05	0.06	1.17	0.10
CCL26	1.00	0.13	0.98	0.10	1.14	0.08	1.21	0.10
CCL28	1.00	0.21	1.09	0.23	1.05	0.25	1.12	0.10
CKB 8-1	1.00	0.15	0.94	0.06	1.06	0.07	1.23	0.11
CNTF	1.00	0.14	0.92	0.08	1.11	0.08	1.17	0.10
CSF2	1.00	0.19	1.09	0.27	1.55	0.27	1.54	0.41
CSF3	1.00	0.28	1.00	0.22	1.06	0.30	1.18	0.23
CTACK	1.00	0.22	0.95	0.22	0.92	0.21	1.21	0.13
CX3CL1	1.00	0.17	0.93	0.08	1.05	0.06	1.18	0.10
Dtk	1.00	0.21	0.95	0.21	0.93	0.14	1.16	0.06
EGF	1.00	0.16	0.92	0.05	1.06	0.06	1.18	0.09
EGFR	1.00	0.22	1.05	0.15	0.95	0.13	1.17	0.11
ENA-78	1.00	0.23	1.13	0.32	1.17	0.41	2.63	1.01
Fas	1.00	0.38	1.09	0.35	1.23	0.48	1.12	0.31
FGF4	1.00	0.25	0.97	0.19	0.95	0.25	1.12	0.12
FGF6	1.00	0.14	0.98	0.10	1.10	0.07	1.13	0.13
FGF7	1.00	0.15	0.89	0.08	1.00	0.08	1.13	0.09
FGF9	1.00	0.19	0.93	0.22	1.01	0.20	1.04	0.10
Flt-3L	1.00	0.15	0.96	0.10	1.06	0.06	1.20	0.12
GCP-2	1.00	0.16	0.99	0.05	1.10	0.08	1.12	0.11
GDNF	1.00	0.17	0.95	0.07	1.16	0.10	1.24	0.10
GITR	1.00	0.18	0.87	0.14	0.87	0.21	1.04	0.11
GITRL	1.00	0.21	0.99	0.18	0.93	0.08	1.06	0.09
GRO	1.00	0.20	1.71	0.79	9.12	5.16	19.09	10.13
GRO-a	1.00	0.24	1.06	0.25	1.10	0.35	1.22	0.23
HCC-4	1.00	0.16	0.96	0.17	0.97	0.20	1.12	0.12
HGF	1.00	0.24	1.26	0.28	0.97	0.13	1.11	0.16
I-309	1.00	0.15	0.94	0.07	1.04	0.06	1.13	0.05
I-TAC	1.00	0.24	0.94	0.23	1.35	0.46	1.13	0.17
ICAM1	1.00	0.26	1.04	0.18	0.98	0.19	1.17	0.09
ICAM3	1.00	0.20	0.94	0.13	0.86	0.13	1.01	0.08
IFN-g	1.00	0.20	0.94	0.31	1.14	0.27	1.21	0.33
IGF-1	1.00	0.15	0.91	0.09	1.04	0.10	1.21	0.08
IGFBP1	1.00	0.16	0.92	0.04	1.01	0.04	1.16	0.12
IGFBP2	1.00	0.19	0.91	0.07	1.12	0.16	1.13	0.08
IGFBP4	1.00	0.16	0.99	0.08	1.12	0.09	1.22	0.09
IGFBP6	1.00	0.22	2.00	0.60	1.23	0.40	1.77	0.56
IGFPB3	1.00	0.21	1.02	0.13	0.90	0.16	1.11	0.12
IL-1 R1	1.00	0.25	0.93	0.22	1.05	0.12	1.11	0.11
IL-10	1.00	0.14	0.93	0.11	1.11	0.08	1.21	0.10
IL-11	1.00	0.27	0.83	0.20	0.90	0.13	0.92	0.13

Cytokine	C-SKMC-S-CM		C-SKMC-T-CM		TNF-SKMC-S-CM		TNF-SKMC-T-CM	
	Protein level (fold C-SKMC-S-CM)	ESM (±)	Protein level (fold C-SKMC-S-CM)	ESM (±)	Protein level (fold C-SKMC-S-CM)	ESM (±)	Protein level (fold C-SKMC-S-CM)	ESM (±)
IL-12 p40	1.00	0.17	1.05	0.16	0.96	0.18	1.01	0.13
IL-12 p70	1.00	0.21	1.00	0.14	0.91	0.18	1.04	0.15
IL-13	1.00	0.06	0.98	0.17	1.12	0.09	1.25	0.17
IL-15	1.00	0.14	0.86	0.10	1.05	0.08	1.18	0.11
IL-16	1.00	0.17	0.89	0.09	1.02	0.06	1.14	0.07
IL-17	1.00	0.22	1.04	0.13	1.04	0.20	1.11	0.12
IL-1a	1.00	0.12	0.97	0.16	1.22	0.08	1.21	0.11
IL-1b	1.00	0.12	0.84	0.09	1.12	0.07	1.09	0.12
IL-1ra	1.00	0.16	0.88	0.10	1.10	0.09	1.17	0.10
IL-2	1.00	0.13	0.84	0.09	1.12	0.07	1.14	0.14
IL-2 Ra	1.00	0.22	0.99	0.19	0.99	0.21	1.20	0.10
IL-3	1.00	0.16	0.99	0.11	1.23	0.04	1.30	0.11
IL-4	1.00	0.15	0.90	0.10	1.07	0.06	1.16	0.07
IL-5	1.00	0.18	0.83	0.11	1.00	0.09	1.10	0.07
IL-6	1.00	0.08	4.92	3.49	14.56	7.47	20.38	9.55
IL-7	1.00	0.16	1.03	0.13	1.03	0.08	1.26	0.10
IL-8	1.00	0.21	1.18	0.35	2.13	1.24	3.29	1.51
IL1R4	1.00	0.24	1.08	0.25	1.17	0.33	1.27	0.39
Leptin	1.00	0.15	0.85	0.05	1.00	0.18	1.25	0.19
LIGHT	1.00	0.15	1.02	0.20	1.08	0.08	1.16	0.09
M-CSF	1.00	0.13	0.93	0.06	1.06	0.07	1.10	0.07
MCP1	1.00	0.34	1.19	0.54	3.12	0.63	3.00	0.48
MCP2	1.00	0.15	1.03	0.24	1.66	0.39	2.35	0.80
MCP3	1.00	0.18	1.05	0.29	7.96	3.85	9.81	4.32
MCP4	1.00	0.16	0.96	0.08	1.05	0.06	1.12	0.09
MDC	1.00	0.19	0.88	0.07	1.00	0.06	1.12	0.09
MIF	1.00	0.30	1.76	0.61	0.96	0.33	1.23	0.38
MIG	1.00	0.15	0.85	0.12	1.31	0.03	1.17	0.10
MIP-1a	1.00	0.21	1.07	0.14	0.90	0.15	1.21	0.07
MIP-1b	1.00	0.23	1.02	0.14	0.82	0.15	1.09	0.11
MIP-1d	1.00	0.14	0.98	0.07	1.10	0.08	1.14	0.08
MIP-3a	1.00	0.17	1.11	0.27	1.31	0.26	2.49	0.81
MIP-3b	1.00	0.18	0.97	0.16	0.89	0.11	1.00	0.17
MSP-a	1.00	0.21	0.97	0.18	0.92	0.21	1.02	0.14
NAP-2	1.00	0.12	1.00	0.04	1.07	0.07	1.42	0.21
NT-3	1.00	0.17	0.93	0.06	1.03	0.07	1.12	0.08
NT-4	1.00	0.31	0.92	0.16	0.87	0.15	0.90	0.10
OPG	1.00	0.39	2.31	0.54	1.45	0.22	5.44	1.38
OSM	1.00	0.16	0.81	0.08	0.81	0.15	0.78	0.10
PARC	1.00	0.15	0.95	0.06	1.06	0.08	1.16	0.07
PDGF-BB	1.00	0.14	0.86	0.09	1.01	0.06	1.12	0.09
PLGF	1.00	0.20	1.01	0.14	0.93	0.18	1.06	0.18
**RANTES**	1.00	0.18	1.03	0.28	24.27	3.43	27.75	2.13
SCF	1.00	0.20	0.91	0.08	0.97	0.06	1.10	0.08
SDF-1	1.00	0.15	0.85	0.10	1.03	0.06	1.14	0.08
sgp130	1.00	0.22	0.99	0.16	0.94	0.20	1.08	0.11
sIGF-1R	1.00	0.17	0.98	0.13	0.81	0.11	1.01	0.13
sIL-6R	1.00	0.22	0.94	0.20	1.00	0.18	1.12	0.09
sTNFR1	1.00	0.23	0.98	0.19	0.90	0.19	1.08	0.09
sTNFR2	1.00	0.24	1.06	0.12	0.88	0.19	1.07	0.13
TARC	1.00	0.15	0.96	0.06	1.02	0.08	1.17	0.12
TECK	1.00	0.21	1.01	0.24	0.91	0.16	1.15	0.10
TGF-b1	1.00	0.12	0.95	0.12	1.10	0.05	1.19	0.12
TGF-b3	1.00	0.13	1.00	0.04	1.07	0.08	1.20	0.08
THPO	1.00	0.23	1.00	0.14	0.89	0.22	1.07	0.12
TIMP1	1.00	0.40	1.36	0.56	1.30	0.63	1.49	0.76
TIMP2	1.00	0.38	1.31	0.38	1.17	0.41	1.18	0.34
**TNF-a**	1.00	0.12	1.17	0.23	10.35	4.21	10.19	4.01
TRAIL R3	1.00	0.24	1.00	0.17	0.87	0.12	1.09	0.11
TRAIL R4	1.00	0.31	1.03	0.20	0.95	0.22	1.03	0.19
uPAR	1.00	0.23	1.00	0.22	1.07	0.16	1.14	0.17
VEGF-A	1.00	0.24	0.82	0.17	0.88	0.13	0.95	0.07
VEGF-D	1.00	0.20	0.96	0.17	0.93	0.16	0.96	0.16
XCL1	1.00	0.18	0.93	0.17	0.88	0.13	1.11	0.11

Table S3: Muscle biopsies donors informations.

	MEAN	SEM
**Age**	22.79	0.38
**Height**	182.79	1.71
**Weight**	75.96	1.89
**BMI**	22.71	0.39
**P**	51.36	1.84
**S-BP (mmHg)**	135.71	1.87
**D-BP (mmHg)**	78.43	1.62
**Hb**	9.10	0.16
**Hct**	0.43	0.01
**Leuco**	5.67	0.36
**Trombo**	214.21	10.42
**Crp**	1.43	0.43
**Na**	142.50	0.57
**K**	3.91	0.05
**TG**	0.89	0.11
**Total chol**	4.15	0.21
**HDL**	1.44	0.06
**LDL**	2.34	0.18
**Creatinin**	82.64	2.68
**Carbamid**	5.24	0.43
**ALAT**	18.18	1.51
**Bilirubin**	13.36	1.91
**Alcalic phos**	61.43	4.52
**F II+VII+X**	0.66	0.02
**HbA1c**	5.11	0.05
**TSH**	2.13	0.22
